# Divergent genetic architecture of cold stress tolerance in *aus* and *tropical japonica* rice

**DOI:** 10.3389/fpls.2025.1716845

**Published:** 2026-01-07

**Authors:** Georgia C. Eizenga, Yami Santamaria, Aaron K. Jackson, Huy Phan, Melissa H. Jia, Quynh P. - H. Grunden, Jeremy D. Edwards, Ed Himelblau, Michael R. Schläppi

**Affiliations:** 1 Dale Bumpers National Rice Research Center, US Department of Agriculture, Agricultural Research Service (USDA-ARS), Stuttgart, AR, United States; 2 Department of Biological Sciences, Marquette University, Milwaukee, WI, United States

**Keywords:** *aus* rice, chilling tolerance, heading date, *Oryza sativa*, rice, plant height, QTL mapping, *tropical japonica* rice

## Abstract

**Introduction:**

Exploring natural genetic variation to facilitate breeding of improved rice seedling cold tolerance will allow the crop to be planted earlier in the growing season, taking advantage of spring rainfall and decreasing exposure to high summer nighttime temperatures, which reduce grain quality.

**Methods:**

To uncover genomic regions in rice that manage cold stress tolerance response mechanisms in the cold-sensitive *aus* (AUS) and the relatively cold-tolerant *tropical japonica* (TRJ) subpopulations, and to identify cold tolerance genes, AUS and TRJ recombinant inbred line populations developed from crosses between cold-tolerant and cold-sensitive parents were used for quantitative trait locus (QTL) mapping of two traits: degree of membrane damage after 1 week of cold exposure, quantified as percent electrolyte leakage (EL), and percent low-temperature seedling survivability (LTSS) after 1 week of recovery growth.

**Results and discussion:**

Thirteen subpopulation-specfic QTL were revealed: three EL and four LTSS QTL for AUS, and two EL and four LTSS QTL for TRJ, with no overlap between AUS and TRJ QTL. Only two AUS QTL overlapped with regions previously identified by our AUS × *temperate japonica* biparental mapping, further confirming the discovery of subpopulation-specific QTL. Based on high-impact genomic differences between the cold-tolerant and cold-sensitive parents, 35 cold tolerance candidate genes were identified—23 in AUS and 12 in TRJ—of which about 50% encode proteins involved in signal transduction and protein homeostasis processes. Although most QTL showed that alleles from cold-tolerant parents improved the two cold tolerance traits, alleles from cold-sensitive parents enhanced these traits at several other QTL. Therefore, alleles from both cold-tolerant and cold-sensitive parents can be used in breeding efforts to generate AUS and TRJ lines with better cold tolerance potential than their respective cold-tolerant parents.

## Introduction

Cultivated Asian rice (*Oryza sativa* L.) is grown worldwide, in diverse environments and on every continent except Antarctica. Rice has two geographically, genetically, and phenotypically diverged varietal groups (VG), *Indica* and *Japonica*. The *Indica* VG is composed of *indica* and *aus* (AUS) cultivars, and the *Japonica* VG is composed of *tropical* (TRJ) and *temperate* (TEJ) *japonica* cultivars. It is generally accepted that TEJ rice cultivars, which predominate in temperate climates including northern China, Korea, and Japan, are more tolerant to cold and chilling temperatures than *Indica* cultivars, which grow in tropical and subtropical regions like southern China and South Asia (Li et al., 2022; Lu et al., 2014; Lv et al., 2015; Mackill and Lei, 1997; Shakiba et al., 2017).

Improving cold tolerance in rice at the seedling stage will increase the crop’s resilience to extreme temperatures, as lower temperatures often lead to poor germination and sterility, which reduce yield (Li et al., 2022). Enhanced tolerance in temperate regions, such as the US Mid-South, will improve seedling vigor, allowing rice to be planted a few weeks earlier without the risk of poor stand establishment and reducing production costs by taking advantage of spring rains. Planting rice earlier in the growing season allows the grain to mature sooner, reducing exposure to high nighttime temperatures during the hottest summer months. Such temperatures (around 30°C) increase grain fissuring (grain cracking), resulting in lower whole-kernel yield—commonly referred to as head rice yield—and reduced grain quality (Gann et al., 2021; Nagata et al., 2013).

Cold tolerance in rice is a multigenic and complex quantitative trait because, depending on temperature, humidity, and photoperiod, different cold stress tolerance response mechanisms are employed by the rice genome through various modules of coexpressed genes (reviewed by Li et al., 2022). As a result, individual genes generally have small effects and act in concert with other genes to protect rice plants from detrimental low-temperature effects. Since 2001, more than 30 studies using traditional biparental linkage mapping projects have identified over 80 germination/seedling stage and approximately 75 reproductive stage quantitative trait loci (QTL), including work from our labs (Schläppi et al., 2023). In addition, over 11 studies since 2015, including four from our labs (Shakiba et al., 2017; Schläppi et al., 2017; Shimoyama et al., 2020; Phan and Schläppi, 2021), using genome-wide association studies (GWAS), have identified at least 435 germination/seedling stage and 65 reproductive stage QTL (reviewed in Li et al., 2022). For breeding purposes, combining different QTL mapping approaches is beneficial to select chromosomal regions consistently uncovered and to identify candidate genes associated with cold tolerance.

To dissect cold tolerance in rice at the germination and seedling stages, Schläppi et al. (2017) developed five assays. One assay evaluated the germination stage, one assay evaluated both the germination and seedling stages, and three assays evaluated the seedling stage. Based on these assays, five cold tolerance indices were calculated and assessed using 202 *O. sativa* accessions from the Rice Mini-Core (RMC) collection. The RMC includes accessions representing the five major rice subpopulations, AUS, *indica*, TRJ, TEJ, and *aromatic* (Sweeney and McCouch, 2007). For young seedling stage indices, the *Japonica* VG and *Indica* VG clustered into cold-tolerant and cold-sensitive accessions, respectively. From this study, the percent low-temperature seedling survivability (LTSS) assay was determined to be the most reliable measure of seedling cold tolerance.

Further investigation of the rice cold tolerance at the seedling stage used a subset of 354 *O. sativa* accessions from the Rice Diversity Panel 1 (RDP1), including representatives of the five rice subpopulations as well as accessions that were admixtures of two or more subpopulations (Shimoyama et al., 2020). These accessions were evaluated for low-temperature tolerance at the seedling stage using three indices: LTSS, percent electrolyte leakage (EL) as a measure of plasma membrane integrity after cold exposure, and median lethal cold temperature (LT50), the temperature at which 50% of 2-week-old seedlings die. Overall, these indices confirmed that accessions in the *Japonica* VG, including both TEJ and TRJ, were cold tolerant; accessions in the *Indica* VG were cold sensitive; and *aromatic* accessions exhibited intermediate cold tolerance.

Mapping cold tolerance traits in both the RMC (Schläppi et al., 2017) and the RDP1 (Shimoyama et al., 2020) using GWAS identified 37 QTL in the RMC and 245 QTL in the RDP1 associated with tolerance. To refine these results and identify smaller genomic regions overlapping with the larger genomic regions detected by GWAS, Schläppi et al. (2023) developed two mapping populations from crosses between two cold-tolerant TEJ accessions and one cold-sensitive AUS accession. Mapping in these populations revealed 16 QTL that overlapped with at least one of the QTL identified by GWAS. The genomic regions delineated by these QTL contained at least 25 candidate genes with polymorphisms between the cold-tolerant TEJ parents and the cold-sensitive AUS parent.

The main objective of this study was to unravel the cold tolerance mechanisms within the AUS and TRJ rice subpopulations and identify subpopulation-specific mechanisms that could be used to improve low-temperature tolerance. To achieve this, we (1) used the RMC LTSS data (Schläppi et al., 2017) to select AUS and TRJ accessions that were either cold tolerant or cold sensitive for developing AUS and TRJ subpopulation-specific biparental recombinant inbred line (RIL) mapping populations; (2) screened the RILs for chilling tolerance and conducted QTL mapping within the populations; (3) identified overlaps with QTL from GWAS mapping; and (4) discovered candidate genes underlying subpopulation-specific chilling tolerance.

## Materials and methods

### Development of the AUS and TRJ subpopulation-specific RIL populations

To develop subpopulation-specific AUS and TRJ biparental RIL populations, the LTSS indices of the *O. sativa* accessions categorized as AUS and TRJ in the RMC (Schläppi et al., 2017) were examined to identify the two most tolerant and two most susceptible accessions in each subpopulation. Seeds of the eight selected RMC accessions were obtained from the Genetic Stocks-*Oryza* (GSOR) collection (https://www.ars.usda.gov/GSOR) at the USDA-ARS Dale Bumpers National Rice Research Center (Stuttgart, AR, USA). Crosses were made between the susceptible and tolerant accessions within each subpopulation, and two to eight *F*_1_ plants from the successful crosses were grown to produce *F*_2_ progeny seeds. Leaf tissue was collected from the *F*_1_ hybrids in spring 2018 to validate their parents using 10 simple sequence repeats (SSR) markers described in Ricebase (https://ricebase.org; Edwards et al., 2016), selected from the original 128 SSR markers, three InDel markers, and six SNP markers used to genotype the RMC (Li et al., 2010). Based on the confirmed parents of the *F*_1_ hybrids, the number of days to 50% heading of the parents, the seed of the parents not having red pericarp, the number of *F*_2_ seeds, plant morphology, and the parents having resequencing data (Wang et al., 2016), the NC 1/536 (GSOR 310381) × Phudugey (GSOR 310715) cross was selected for developing the AUS subpopulation-specific RIL population, and the Taichu Mochi 59 (GSOR 310080) × British Honduras Creole (GSOR 310144) cross for developing the TRJ subpopulation-specific RIL populations.

For the AUS population, NC 1/536, the cold-susceptible female parent, originated from Pakistan and had a mean LTSS at 10°C of 1.54% (Schläppi et al., 2017). Phudugey, the cold-tolerant male parent, originated from Bhutan and had a mean LTSS at 10°C of 37.42% (Schläppi et al., 2017). Four (RM234, RM228, RM224, and RM154) of the 10 SSR markers were used to confirm these *F*_1_ hybrids, and the genetic distance between the AUS parents is shown in **Supplementary Figure S1**. To develop the NC 1/536 × Phudugey RIL population, about 220 single *F*_2_ seeds were planted in the greenhouse near Stuttgart, AR, USA, to produce *F*_3_ seeds. Using the single-seed descent method, all *F*_2_ plants that produced *F*_3_ seeds were advanced to the *F*_4_, *F*_5_, and *F*_6_ generations. For genotyping, leaf tissue was collected from 168 individual *F*_6_ plants grown in the greenhouse (for three RILs missing tissue, tissue from *F*_7_ field plants was used for genotyping). The *F*_7_ seeds harvested from these plants were planted in the field near Stuttgart, AR, USA, on 4 May 2022 to produce adequate seed for phenotyping the population for cold tolerance at the seedling stage (11 RILs did not produce an *F*_7_ seed; thus, an *F*_6_ seed was planted). Data on agronomic traits were collected from 153 *F*_6:7_ plants (described below), and *F*_7:8_ seed was collected from a single *F*_6:7_ plant of each of the 152 RILs for conducting the cold tolerance phenotyping (**Supplementary Table S1**) (the aus_197 RIL did not produce an *F*_8_ seed for the cold tolerance phenotyping).

For the TRJ population, Taichu Mochi 59, the cold-susceptible female parent, originated from Taiwan and had a mean LTSS at 10°C of 39.76% (Schläppi et al., 2017). British Honduras Creole, the cold-tolerant male parent, originated from Belize and had a mean LTSS at 10°C of 92.14% (Schläppi et al., 2017). Eight (RM234, RM190, RM215, RM1339, RM224, RM154, RM232, and RM5) of the 10 SSR markers were used to confirm the *F*_1_ hybrids, and the genetic distance between these TRJ parents is shown in **Supplementary Figure S1**. To develop the Taichu Mochi 59 × British Honduras Creole TRJ RIL population, 200 *F*_2_ seeds were planted in the greenhouse to produce *F*_3_ seeds. Using the single-seed descent method, all *F*_2_ plants that produced *F*_3_ seeds were advanced to the *F*_4_, *F*_5_, and *F*_6_ generations. For genotyping, leaf tissue was collected from 144 individual *F*_6:7_ plants grown in the greenhouse, and the seeds harvested from 140 TRJ RILs were planted in the field to produce adequate seeds for phenotyping the population for cold tolerance at the seedling stage (**Supplementary Table S2**; two RILs did not produce seeds, so *F*_5_ seeds were planted in the field). Data on agronomic traits were collected from 122 *F*_5:7_ plants (described below). Note that the trj_35 RIL seed did not germinate in the field, so it was grown in the greenhouse; thus, no agronomic data were recorded. For cold tolerance phenotyping, *F*_6:8_ seeds were collected from a single plant of 123 TRJ RILs, and 90 of these RILs were used for the cold phenotyping.

### Phenotyping of the AUS and TRJ RIL populations

#### Days to heading, plant height, and culm habit

The AUS and TRJ populations were grown under field conditions to phenotypically characterize the RILs for important agronomic traits, including days to 50% heading (HD), plant height (PTHT), and culm habit (CULMHAB), and to determine which candidate genes were potentially present in the populations. Field conditions also allowed the production of larger amounts of seed to phenotype the populations for seedling cold tolerance traits. Thus, seeds of 168 AUS RILs and 140 TRJ RILs were planted in the field near Stuttgart, AR, USA, on 4 May 2022. For each RIL, eight *F*_6:7_ seeds were planted in a row, with 30.5 cm between plants within the row and 198 cm between rows. The number of seeds that germinated per row ranged from zero to eight plants, with an average of 3.3 plants per AUS RIL row and 3.2 plants per TRJ RIL row. The RILs were planted, one replication each, in two bays, with the four parents serving as the outer rows per tier: NC 1/536 and Phudugey for the AUS RILs, and Taichu Mochi 59 and British Honduras Creole for the TRJ RILs. Emergence occurred on 19 May 2022, and the bays were fertilized and flooded on 14 June. Seeds were harvested in mid-September, between 12 and 20 September 2022.

The RILs and parents were characterized for three agronomic traits. HD was determined as the number of days from planting until about 50% of the panicles in a row reached anthesis. For PTHT and CULMHAB, each row of an individual RIL was observed, and based on uniformity, one representative plant was selected. Plant height was measured from the soil surface to the tip of the longest panicle at harvest. Culm habit was rated as erect (1), intermediate (3), open (5), spreading (7), or prostrate (9) after observing all plants in the row. Panicles from the phenotyped RIL were harvested, and the resulting seed was used for cold tolerance tests.

**Supplementary Figure S2A** presents images of the AUS RIL parents, NC 1/536 and Phudugey, and a selected AUS RIL. Most of the AUS RILs were lodged due to being tall plants and having weak tillers; therefore, images were not recorded. **Supplementary Figure S2B** includes images of the TRJ RIL parents, Taichu Mochi 59 and British Honduras Creole, and selected TRJ RILs.

#### Germination and standard seedling growth conditions

For cold phenotyping, seeds of the four parents and RILs were germinated in the dark for 2 days at 37°C in deionized water containing 0.1% bleach to prevent bacterial contamination. Germinating seeds were transferred into PCR strips, placed into pipette tip boxes, and grown hydroponically in deionized water for 10 days in a growth chamber under 12-h light (approximately 150 µE photon flux)/12-h dark cycles, with day/night temperatures of 28°C/25°C. On day 10, the water was replaced with one-fourth Murashige–Skoog basal salt liquid medium to provide nutrients. Each line was represented by up to eight plants per box in quadruplicate, for a maximum of 32 plants (four boxes of eight plants) per experiment. The four boxes were randomly arranged within the growth chamber. Each box contained 11 strips of RILs and one strip containing four seedlings of each parent as controls. For the AUS RILs, there were four NC 1/536 seedlings, the cold-sensitive control, and four Phudugey seedlings, the cold-tolerant control. For the TRJ RILs, the cold-tolerant parent, British Honduras Creole, and the relatively cold-sensitive parent, Taichu Mochi 59, were used as controls.

#### Chilling stress treatment

Four boxes containing 2-week-old seedlings at the two-leaf stage were placed at random positions within a growth chamber maintained at a constant 10°C ± 1°C and incubated for 7 days (12-h light/12-h dark cycles). The seedlings were watered every other day.

#### Electrolyte leakage

At the end of the 7-day 10°C stress period, the middle section of the second leaf from three individual seedlings per RIL or control per box was collected and cut into three equally sized segments. The pieces were washed in deionized water and transferred into three different screw-cap glass tubes filled with 5 ml of deionized water (conductivity ≤ 2 µS/cm), then shaken at 200 rpm for 60 min at room temperature to release cellular electrolytes from low-temperature-damaged tissues. Initial conductivity of the three replicates per box (a total of 12 replicates across the four randomly distributed boxes) was measured by taking 120 µl of the solution and adding it to the well of a handheld LAQUAtwin B-771 conductivity meter (Horiba Scientific, Kyoto, Japan). Leaf samples were boiled for 20 min after the initial reading to release total cellular electrolytes. Samples were shaken again at 200 rpm for 30 min after cooling to room temperature, and the final conductivity reading was taken. Percent EL for each sample was determined as [(initial conductivity reading)/(final conductivity reading)] × 100.

#### Low-temperature seedling survivability

At the end of the 7-day 10°C stress period, seedlings were returned to standard growth conditions for 1 week to recover, after which seedling survival was assessed visually. Seedlings that were mostly green and stiff were scored as alive, whereas seedlings that were mostly wilted and/or bleached and soft were scored as dead. The mean percent survivability was calculated as [(number of seedlings scored as alive)/(total number of stressed plants)] × 100.

### Statistical analysis

Both the percent EL and percent LTSS were calculated using a linear mixed model (LMM) to obtain best linear unbiased predictions (BLUPs). The LMM used an augmented design with a fixed-effect variable (group) based on whether the line was a control or RIL. The line nested within the group variable, set (growth chamber and experiment date), and the box were assigned as random effects. In addition to percent LTSS (useful for direct comparison to previous studies), a more appropriate statistical method for survival analysis was employed. Specifically, a binomial generalized linear mixed model (GLMM) with a logit link function was used to predict the probability of survival under cold treatment on the log-odds scale. The same fixed and random variables were used for all traits in both the GLMM and LMM models.

All calculations were performed using JMP 18 (JMP 18.2.0, 2022), including the mean and standard error of the mean for each of the six traits across the AUS and TRJ RILs. Frequency distribution images for the AUS and TRJ RILs for each trait were generated in JMP. Correlations among the six traits were calculated using the multivariate option with the restricted maximum likelihood (REML) method, and the corresponding scatterplot matrix was also produced in JMP.

### Genotyping and QTL mapping

Leaf tissue was collected from the parents and progeny of both *F*_6:7_ RIL populations and lyophilized. Genotyping was performed with the Cornell-IR LD Rice Array (C7 AIR) with 7,098 SNP markers (Morales et al., 2020) available as an Illumina Infinium array. Lyophilized leaf tissues were sent to Eurofins Diagnostics Inc. (www.eurofinsgenomics.eu/en/genotyping-gene-expression/service-platforms/illuminaarrayplatforms/) for DNA extraction and sequencing. Genotypes were filtered to remove markers or lines that were monomorphic, had 30% or greater missing data, had excessive heterozygous calls, or did not appear to map correctly. For each population, all available genotyped lines were used to create a genetic linkage map in MapDisto version 1.7.7 (Lorieux, 2012); lines missing all three traits from cold tolerance traits or agronomic traits were then removed for mapping purposes.

The AUS RIL population had 519 SNPs with 152 RILs used for mapping cold tolerance traits (percent electrolyte leakage, percent low temperature seedling survivability, and low temperature seedling survivability logit) and 153 progeny lines used to map agronomic traits (DH, PTHT, and CULMHAB). The TRJ RIL population had 1,042 SNPs, with 90 RILs used to map cold tolerance traits and 122 RILs used to map agronomic traits. Parents of the respective mapping populations were included in the QTL analysis. QTL mapping was performed in Qgene version 4.4.0 (Joehanes and Nelson, 2008) using single-trait multiple interval mapping, with the scan size set at 0.1 centimorgans (cM) for both populations. For each trait, a permutation analysis was performed in Qgene using the default setting of 1,000 permutations and alpha levels of 0.01, 0.05, and 0.10. For comparison, QTL mapping of both populations was also conducted in IciMapping software version 4.2 (Integrated Software for Building Genetic Linkage Maps and Mapping Quantitative Trait Loci; Meng et al., 2015) using default settings for Inclusive Composite Interval Mapping (ICIM). One thousand permutations were used to test each trait at a significance level of 0.05 to determine LOD thresholds.

### Annotation of candidate genes

Candidate genes were manually identified within 1.5 Mb on each side of the reported QTL peak by searching a master file compiled from multiple databases and manuscripts, as described in Huggins et al. (2019). The file was updated in May 2024 and includes gene annotations from the Os-Nipponbare-Reference _IRGSP-1.0 assembly (https://rice.uga.edu/pub/data/Eukaryotic_Projects/o_sativa/annotation_dbs/; accessed 28 May 2024) (Kawahara et al., 2013; Ouyang et al., 2006), the Rice Annotation Project (RAP-Db, https://rapdb.dna.affrc.go.jp/download/irgsp1.html?version=2024-01-11; accessed 28 May 2024) (Sakai et al., 2013), candidate genes for biotic and abiotic stress identified from Cohen and Leach (2019), genes listed in *Oryzabase* (https://shigen.nig.ac.jp/rice/oryzabase/download/gene; accessed 28 May 2024) (Yamazaki et al., 2010), genes listed in RiceNavi (Wei et al., 2021), and cloned genes from FunRiceGenes (https://funricegenes.github.io/geneInfo.table.txt; accessed 28 May 2024) (Huang et al., 2022).

### *In silico* analysis of candidate genes within mapped QTL

All loci associated with a particular QTL were retrieved from the Rice Annotation Project Database (https://rapdb.dna.affrc.go.jp; Sakai et al., 2013). Genomic variation within these loci was obtained from the RiceVarMap database (https://ricevarmap.ncpgr.cn; Zhao et al., 2015) by identifying polymorphic positions between cultivars (cultivar IDs W025 and W409 for the TRJ-derived QTL, cultivar IDs W005 and W303 for the AUS-derived QTL). Loci containing InDels, nonsense mutations, and/or likely splice site mutations were considered for further analysis, as these variants are likely to cause changes in protein function.

Expression data for these candidate genes were obtained from NCBI project IDs PRJNA610422 and PRJNA430015 using the Rice RNA-seq Database (https://plantrnadb.com/ricerna/; Yu et al., 2022). For PRJNA610422, 4°C-0 h samples and 4°C-12 h samples were compared. For PRJNA430015, “CTR control” samples and “CTR cold stress” samples were compared. A heteroscedastic *t*-test was performed to determine whether expression differed significantly between samples.

## Results and discussion

### Description of AUS and TRJ RIL populations

The NC 1/536 × Phudugey AUS RILs, Taichu Mochi 59 × British Honduras Creole TRJ RILs, and the four parents were phenotyped under field conditions for the agronomic traits, HD, PTHT, and CULMHAB (**Supplementary Figure S2** shows images of the AUS parents and a representative RIL in **Supplementary Figure S2A**, and the TRJ parents with selected RILs in **Supplementary Figure S2B**). In the AUS population, Phudugey, the male parent, headed slightly later and was slightly taller than the female parent, NC 1/536. The 153 RILs exhibited transgressive variation, with progeny taking 93 to 128 days to reach 50% heading, and plant height ranged from 100 to 195 cm (**Table 1**; **Supplementary Table S1**; **Supplementary Figure S3A**). The mean RIL rating for culm habit was open (5.7), similar to both parents, but individual RILs ranged from erect (1) to spreading (7).

**Table 1:** Summary statistics (overall mean, SE, and range of progeny) of six traits measured in the NC 1/536 (GSOR 310381) × Phudugey (GSOR 310715) *aus* (AUS) RIL population included in the quantitative trait locus analysis (**Tables 3**, **4**) and in the parents.

Trait (acronym)	AUS recombinant inbred line progeny^a^			Parents	
	Mean	SE^b^	Range	NC 1/536	Phudugey
Agronomic traits					
Days to 50% heading (HD)	105.9	0.5	93–128	102.8	105.1
Plant height (PTHT) (cm)	156.7	1.1	100–195	153.6	158.9
Culm habit (CULMHAB)^c^	5.7	0.1	1–7	5.6	5.4
Chilling tolerance traits					
Electrolyte leakage (EL) (%)	26.52	0.60	14.60–54.20	25.14	25.22
Low-temperature seedling survivability (LTSS) (%)^d^	58.58	1.67	3.72–97.83	30.93	82.15
Low-temperature seedling survivability (LTSS) (logit)	0.56	0.12	− 4.23 to 3.72	− 1.07	2.00

Trait distributions are shown in **Supplementary Figure S1A**, with the corresponding data provided in **Supplementary Table S1**.

a For the agronomic traits, 153 AUS RILs were evaluated, and 152 RILs were evaluated for the chilling tolerance traits.

b The SE of the mean was calculated as the SD divided by the square root of the number of entries.

c The categories for culm habit are as follows: erect (1), intermediate (3), open (5), spreading (7), and prostrate (9).

d The percentage LTSS for each RIL and parent was calculated as a BLUP-adjusted mean.

The parents of the TRJ population, Taichu Mochi 59 and British Honduras Creole, also differed for the three agronomic traits evaluated. Compared to Taichu Mochi 59, the male parent, British Honduras Creole, took about five fewer days to head, was taller, and had an open (4.4) culm habit compared with the erect (1.4) culm habit of Taichu Mochi 59. The 122 TRJ RILs exhibited transgressive variation for all three agronomic traits, as presented in **Table 2**, **Supplementary Table S2**, and **Supplementary Figure S3B**.

**Table 2:** Summary statistics (overall mean, SE, and range of progeny) of six traits measured in the Taichu Mochi 59 (GSOR 310080) × British Honduras Creole (GSOR 310144) *tropical japonica* (TRJ) RIL population included in the quantitative trait locus analysis (**Tables 3**, **4**) and in the parents.

Trait (acronym)	TRJ recombinant inbred line progeny^a^			Parents	
	Mean	SE^b^	Range	Taichu Mochi 59	British Honduras Creole
Agronomic traits					
Days to 50% heading (HD)	110.1	0.5	90.0–124.0	110.0	104.9
Plant height (PTHT) (cm)	139.2	0.9	110.0–165.0	127.8	147.1
Culm habit (CULMHAB)^c^	3.0	0.2	1–7	1.4	4.4
Chilling tolerance traits					
Electrolyte leakage (EL) (%)	21.62	0.84	4.92–48.72	14.86	29.24
Low-temperature seedling survivability (LTSS) (%)^d^	82.46	1.76	4.77–109.81	66.15	94.72
Low-temperature seedling survivability (LTSS) (logit)	2.66	0.14	− 2.61 to 5.15	1.20	4.12

Trait distributions are shown in **Supplementary Figure S1B**, and the data are in **Supplementary Table S2**.

a For the agronomic traits, 122 TRJ RILs were evaluated, and 90 RILs were evaluated for the chilling tolerance traits.

b The SE of the mean was calculated as the SD divided by the square root of the number of entries.

c The categories for culm habit are as follows: erect (1), intermediate (3), open (5), spreading (7), and prostrate (9).

d The percentage LTSS for each RIL and parent was calculated as a BLUP-adjusted mean; therefore, the percentage exceeds 100% for eight RILs.

Both the AUS and TRJ RIL populations were evaluated for two low-temperature traits: EL, where more cold-tolerant RILs generally exhibit less leakage, and LTSS, where more cold-tolerant RILs have higher survivability. The LTSS values for the RILs were not normally distributed, so the logit function in the GLMM was used to transform the data, resulting in a distribution closer to normal, particularly for the TRJ RILs (**Supplementary Figures S3A, B**). Comparing the distribution of the 152 AUS RILs evaluated for cold tolerance (**Table 1**; **Supplementary Figure S3A**) with that of the 90 TRJ RILs evaluated for the same traits (**Table 2**; **Supplementary Figure S3B**) confirmed that the *Japonica* VG, which includes the TRJ subpopulation, is more cold tolerant than the *Indica* VG, which includes the AUS subpopulation (Liu et al., 2018). This is evident by the lower mean EL in the TRJ RILs (21.62%) compared with the AUS RILs (26.52%). Similarly, the mean LTSS (82.46%) and LTSS-logit value (2.66) for the TRJ RILs were higher than the mean LTSS (58.58%) and LTSS-logit (0.56) value for the AUS RILs.

For the AUS RILs, significant negative correlations were observed between heading date and both plant height (*r* = − 0.32; *p* ≤ 0.0001) and culm habit (*r* = − 0.22; *p* ≤ 0.01), indicating that taller plants took fewer days to head and were more upright (**Supplementary Figure S4A**). Plant height and LTSS BLUP and logit (*r* = 0.26; *p* = 0.01) had significant positive correlations, suggesting that taller RILs had higher LTSS in this population. As expected, EL was negatively correlated with LTSS logit and BLUP (*r* = − 0.28; *p* = 0.001), since EL decreases with increased LTSS. For the TRJ RILs, culm habit had significant negative correlations with heading date (*r* = − 0.48; *p* ≤ 0.001) and both LTSS values, logit (*r* = − 0.27; *p* = 0.01) and BLUP, as shown in **Supplementary Figure S4B**, indicating that more upright TRJ RILs took longer to head and were more cold tolerant. The significant correlation between plant height and LTSS observed in the AUS RILs was not found in the TRJ RILs.

### Mapping potential validation of agronomic traits for AUS and TRJ RIL populations

The 153 NC 1/536 × Phudugey AUS RILs and their parents were genotyped with the 7,098 DNA markers included on the C7AIR (Morales et al., 2020). Of these, 519 markers were polymorphic between the parents, and 516 were used to construct a 416.50-centimorgan (cM) linkage map. The markers mapped to 292 different cM positions, with an average interval of 1.43 cM and a maximum distance of 14.09 cM between markers (**Supplementary Table S3**). Four regions exhibited significant segregation distortion based on *χ*^2^ values. Three regions on chr. 2 (32.6–35.2 cM), chr. 4 (4.0–4.9 cM), and chr. 6 (23.6–33.6 cM) were distorted in favor of the Phudugey alleles, while one region on chr. 9 (22.0–22.5 cM) was distorted in favor of NC 1/536 alleles.

**Figure 1:**
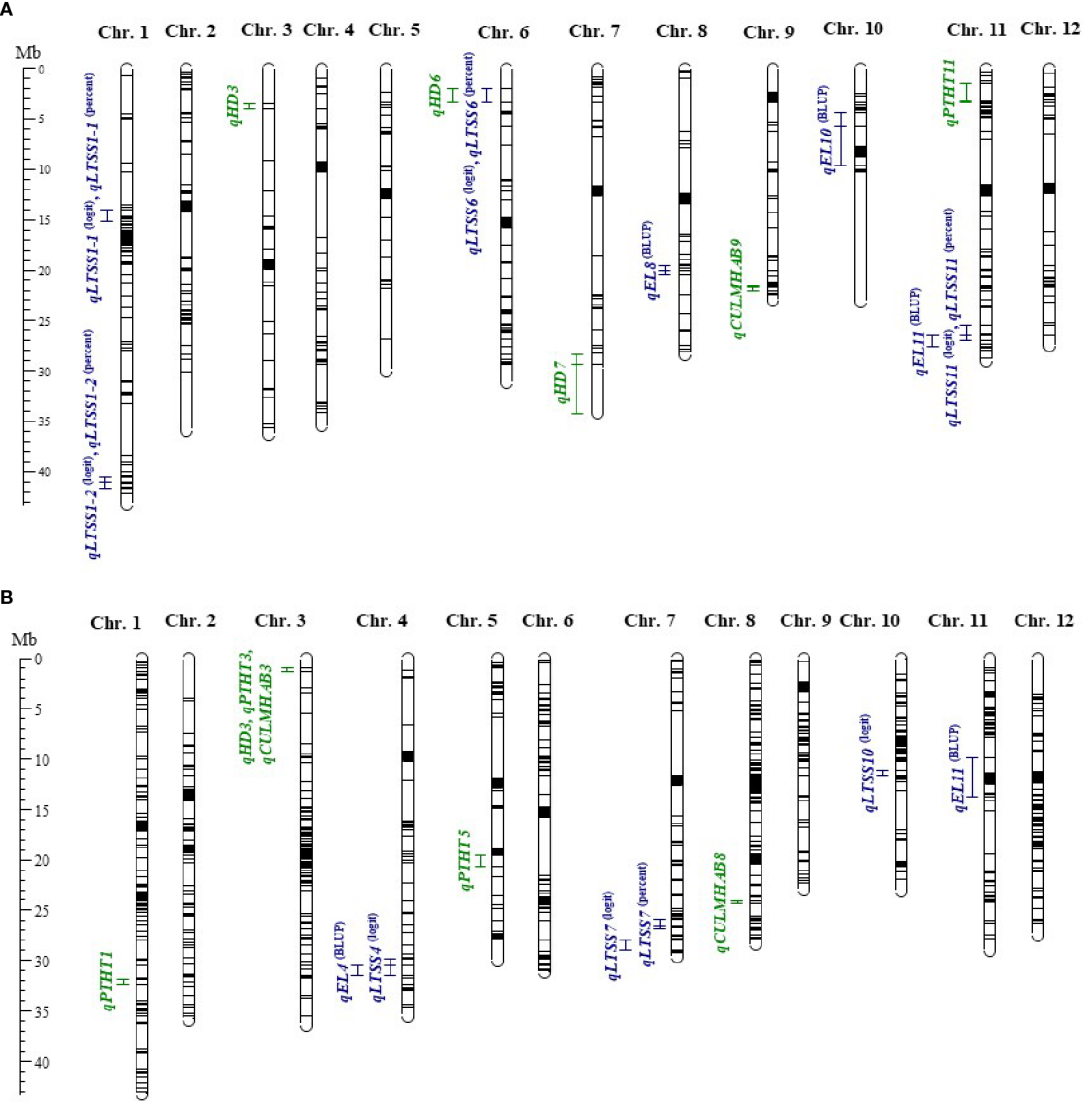
Quantitative trait loci (QTL) maps of **(A)** AUS and **(B)** TRJ recombinant inbred lines (RILs) in Mb. Cold tolerance traits are shown in blue and agronomic traits in green. Three cold tolerance traits were mapped in both panels: percent electrolyte leakage (*qEL*), percent low temperature seedling survivability (*qLTSS*^(percent)^), and low temperature seedling survivability logit (*qLTSS*^(logit)^), which predicts the probability of survival under cold treatment on the log-odds scale. Three agronomic traits were also determined: days to 50% heading (*qHD*), plant height (*qPTHT*), and culm habit (*qCULMHAB*). In **(A)**, the mapping was conducted using NC 1/536 (GSOR 310381) × Phudugey (GSOR 310715) RILS—152 for cold tolerance and 153 for agronomic traits—with 516 single-nucleotide polymorphism (SNP) markers; while in **(B)**, the mapping was conducted using Taichu Mochi 59 (GSOR 310080) × British Honduras Creole (GSOR 310144) RILs—90 for cold tolerance and 122 RILs for agronomic traits—with 1,042 SNP markers. The 12 rice chromosomes are shown with SNP markers as gray horizontal lines and centromeres in black. (Maps created with MapChart 2.3 [Voorrips, 2002].).

QTL mapping was conducted for three agronomic traits in both the AUS and TRJ RIL populations as a “quality control” to assess whether known QTL for these traits and the associated genes could be uncovered (**Table 3**). QTL mapping within the AUS RILs revealed five QTL for agronomic traits: three HD QTL, one PTHT QTL, and one CULMHAB QTL (**Table 3A**; **Figure 1A**). For the three HD QTL—*qHD3*, *qHD6*, and *qHD7*—the additional days were attributed to the Phudugey parent. Of note, these three HD-QTL regions include four well-known genes for heading date, namely *qHD3*, encompassing the *MADS BOX GENE 50* (*MADS50*) at 1.30 Mb on chr. 3 (Lee et al., 2004); *qHD6*, including both *RICE FLOWERING-LOCUS T 1* (*RFT1*) at 2.93 Mb (Komiya et al., 2009) and *HEADING DATE 3A* (*HD3A*) at 2.94 Mb (Kojima et al.., 2002), both on chr. 6; and lastly, *qHD7*, which most likely includes *HEADING DATE* 2 (*HD2*) on chr. 7 at 29.62 Mb, first identified in the late 1990s (Yano et al., 1997; Yamamoto et al., 1998).

**Table 3:** Days to 50% heading (HD), plant height (PTHT), and culm habit (CULMHAB) quantitative trait loci (QTL) identified in the NC 1/536 × Phudugey *aus* (AUS) and Taichu Mochi 59 × British Honduras Creole *tropical japonica* (TRJ) recombinant inbred line (RIL) mapping populations.

QTL^a^	Chr.	QTL region (cM)^b^	QTL interval (Mb)^c^	Marker nearest LOD peak	Peak position (cM)	LOD value^d^	Additive effect^e^	PVE^f^	Gene symbol^g^	Position (Mb)^h^	LOC_ID^i^	Citation
NC 1/536 × Phudugey AUS RIL population												
*qHD3*	3	0.0–0.8	3.5–4.0	3_3542519	0.2	2.66	− 1.47	7.60	*MADS50*	1.30	LOC_Os03g03100	Lee et al. (2004)
*qHD6*	6	0.1–2.8	2.0–3.4	6_2010737	0.0	2.73	− 1.51	7.80	*RFT1*	2.93	LOC_Os06g06300	Komiya et al. (2009)
*qHD7*	7	30.4–34.1	28.3–34.2	7_29288097	32.8	2.97	− 1.65	8.40	*HD2*	29.62	LOC_Os07g49460	Yano et al. (1997); Yamamoto et al. (1998)
*qPTHT11*	11	5.2–6.9	1.4–3.3	11_3244411	6.4	3.53	− 4.18	1.00	*PK1*	2.24	LOC_Os11g05110	Zhang et al. (2012)
*qCULMHAB9*	9	21.5–22.0	21.6–22.1	9_21729544	21.8	2.93	0.39	8.30	*AHP2*	22.65	LOC_Os09g39400	Sun et al. (2014)
Taichu Mochi 59 × British Honduras Creole TRJ RIL population												
*qHD3*	3	0.0–0.6	1.0–1.3	3_1292063	0.6	4.57	2.19	15.60	*MADS50*	1.30	LOC_Os03g03100	Lee et al. (2004)
*qPTHT1*	1	57.0–57.7	31.8–32.3	1_32326843	57.7	3.44	− 3.34	12.00	*GA2OX3*	31.80	LOC_Os01g55240	Sakai et al. (2003)
*qPTHT3*	3	0.0–0.6	1.0–1.3	3_1292063	0.6	3.62	3.34	12.60	*CSN1*	0.94	LOC_Os03g02540	Liu et al. (2025)
*qPTHT5*	5	32.6–34.7	19.5–20.7	5_19504928	32.5	3.12	− 3.50	10.90	*SDF5*	20.33	LOC_Os05g34325	Yachun et al. (2021)
									*OsGDI1*	20.48	LOC_Os05g34540	Shad et al. (2023)
*qCULMHAB3*	3	0.0–0.6	1.0–1.3	3_1292063	0.6	3.53	− 0.61	12.30	*TAD1*	1.33	LOC_Os03g03150	Xu et al. (2012)
*qCULMHAB8*	8	34.2–34.6	24.1–24.3	8_24128013	34.2	4.31	0.70	14.80	*WFP*	25.27	LOC_Os08g39890	Miura et al. (2010); Jiao et al. (2010)

Known candidate genes located in the QTL regions are listed. The AUS RIL population had 153 progeny genotyped with 516 SNP markers, and the TRJ RIL population had 90 progeny genotyped with 1,042 SNP markers.

a Quantitative trait loci (QTL) were declared based on Qgene version 4.4.0 (Joehanes and Nelson, 2008) using single-trait multiple interval mapping with a default scan size of 10 millimorgans (mM). After initial QTL identification for each trait, the scan size was changed to 1 mM, and QTL mapping was rerun to focus on the chromosomes containing the QTL regions.

b The QTL region is defined in centimorgans (cM) and based on the linkage map.

c The QTL interval is defined by the megabase positions of the DNA markers closest to the extremities of the QTL region.

d Across all traits, QTL were declared significant if the LOD value was about 3.5 (α = 0.05, 1,000 permutations). LOD values around 2.7 were considered significant at α = 0.10 with 1,000 permutations.

e A positive additive effect indicates that the NC 1/536 or Taichu Mochi 59 allele increases the phenotype for that trait. A negative additive effect indicates that the Phudugey or British Honduras Creole allele increases the phenotype.

f PVE is the percentage of total phenotypic variation explained by an individual QTL, estimated from *R*^2^ values in the Qgene analysis.

g Gene nomenclature follows the standardized rice gene nomenclature in *Oryzabase* (Yamazaki et al., 2010), using the Committee on Gene Symbolization, Nomenclature, and Linkage gene symbols.

h The physical position of the candidate gene is based on the Rice Genome Annotation Project Release 7.

i Rice Genome Annotation Project locus identifier for the candidate gene (Kawahara et al., 2013).

A single PTHT QTL, *qPH11*, was detected on chr. 11 and was attributed to the Phudugey parent (**Supplementary Table S3**). One candidate gene, *PYRUVATE KINASE 1* (*PK1*) at 2.24 Mb, was identified within this QTL region. Pyruvate kinase (PK) catalyzes the final step of glycolysis and influences the glycolytic pathway, and studies of *OsPK1* mutants have demonstrated its role in plant morphological development, particularly affecting plant height (Zhang et al., 2012).

Similarly, a single QTL for culm habit, *qCULMHAB9*, was detected on chr. 9. One of the two His phosphotransfer proteins (HP), *HISTIDINE CONTAINING PHOSPHOTRANSMITTER 2* (*AHP2*), which regulates cytokinin signaling, is located in this QTL region at 22.65 Mb. Cytokinin is an important phytohormone that regulates plant growth and development, and Sun et al. (2014) reported that plants deficient in cytokinin signaling were shorter due to reduced internodes, had fewer tillers, and reduced fertility, which may have affected culm habit.

The 123 Taichu Mochi 59 × British Honduras Creole TRJ RILs and their parents were genotyped using the 7,098 DNA markers included on the C7AIR (Morales et al., 2020). Of these markers, 1,045 were polymorphic between the parents, and 1,042 were used to construct a 569.2-cM linkage map. The markers were mapped to 512 different cM positions, with an average interval of 1.11 cM and a maximum of 12.10 cM between markers (**Supplementary Table S4**). Six regions exhibited significant segregation distortion based on *χ^2^* values. Four regions on chr. 1 (35.5–44.6 cM), chr. 5 (3.7–23.7 cM), chr. 7 (26.1–31.0 cM), and chr. 8 (30.1–38.7 cM) were distorted in favor of the British Honduras Creole alleles, whereas two regions on chr. 9 (5.7–25.1 cM) and chr. 12 (1.8–9.0 cM) were distorted in favor of Taichu Mochi 59.

QTL mapping in the TRJ population with 122 RILs for agronomic traits revealed six QTL (**Table 3**; **Figure 1B**). One TRJ RIL (trj_35) did not have agronomic data because the line did not grow in the field; thus, it was only used for mapping the cold tolerance traits. Mapping days to HD revealed a single QTL, *qHD3*, which included the well-known *MADS50* gene at 1.30 Mb on chr. 3 (Lee et al., 2004). This QTL was attributed to the Taichu Mochi 59 parent, which had delayed heading. Notably, *MADS50* was also one of the four candidate genes identified with HD QTL mapping in the AUS RIL population.

Three PTHT QTL were found, with *qPTHT1* and *qPTHT5* attributed to the British Honduras Creole parent and *qPTHT3* to Taichu Mochi 59. The *qPTHT1* region (31.8–32.3 Mb) was proximal to the well-known “green revolution gene”, *SEMI-DWARF 1* (*SD1*), on chr. 1 at 38.38 Mb, which produced semidwarf plants due to a mutation in the *GIBBERELLIN 20-OXIDASE2* (*GA20OX2*) gene, resulting in dwarfism caused by a deficiency in the gibberellin (GA) plant growth hormones (Sasaki et al., 2002; Spielmeyer et al., 2002). One candidate gene in the *qPH1* region, *GIBBERELLIN 2-OXIDASE 3* (*GA2OX3*) at 31.80 Mb (Sakai et al., 2003), is involved in the regulation of GA. A second gene in this region, *GRETCHENHAGEN 3-2* (*OsGH3-2*) at 32.46 Mb, encodes an indoleacetic acid-amido synthetase and interacts with the *GA20OX1* and *GA20OX2* genes to affect plant height by regulating GA biosynthesis and auxin metabolism (Kim et al., 2024). Also, in the *qPTHT5* region, *SEMI-DWARF 5* (*SDF5*) is located at 20.33 Mb and is involved in the GA biosynthesis pathway and modifies plant architecture, including plant height (Yachun et al., 2021). In the *qPTHT3* region, the *COP9 SIGNALOSOME COMPLEX SUBUNIT 1* (*CSN1*) was a positive regulator of plant height under far-red light, and exogenous GA_3_ treatment increased height (Li et al., 2025). Lastly, single mutations in the *GDP DISSOCIATION INHIBITOR 1* (*OsGDI1*) in the *qPTHT5* region resulted in dwarf plants as well as longer and thinner grains (Shad et al., 2023).

Culm habit (*CULMHAB*) QTL were discovered on chr. 3, *qCULMHAB3*, with the increased spreading attributed to British Honduras Creole, and chr. 8, *qCULMHAB8*, with the increase attributed to Taichu Mochi 59. Genes associated with plant architecture were identified in or near each of these QTL regions. *TILLERING AND DWARF 1* (*TAD1*) is on chr. 3 at 1.33 Mb (Xu et al., 2012) mainly affects tiller number and plant height. The *WEALTHY FARMER’S PANICLE* (*WFP*) or *IDEAL PLANT ARCHITECTURE 1* (*IPA1*) is on chr. 8 at 25.27 Mb (Miura et al., 2010; Jiao et al., 2010) and encodes *SOUAMOSA PROMOTER BINDING PROTEIN-LIKE 14* (*OsSPL14*), which affects plant height, tiller number and angle, panicle branching, and the number of grains produced.

### Cold tolerance trait mapping

QTL mapping was conducted for two low-temperature traits in both the AUS and TRJ RIL subpopulations. Percent EL was used to measure membrane damage right after 1 week of exposure to 10°C, and percent LTSS was used to measure seedling survival after 1 week of exposure to 10°C and 1 week of recovery at warm temperatures. This revealed three EL QTL (*qEL*) and four LTSS QTL (*qLTSS*) for the AUS subpopulation, and two *qEL* and four *qLTSS* for the TRJ subpopulation (**Table 4**). For *qLTSS* mapping in the AUS subpopulation, increased seedling survivability associated with three of the four LTSS QTL—*qLTSS1–1*, *qLTSS6*, and *qLTSS11*—was attributed to alleles from the relatively cold-tolerant Phudugey parent, while for *qEL* mapping, increased electrolyte leakage and thus increased membrane damage after cold exposure associated with two of the three EL QTL, *qEL8* and *qEL11* (overlapping with *qLTSS11*), was attributed to alleles from the cold-sensitive NC 1/536 parent. This showed that although the cold-tolerant parent contributed more alleles to enhance cold tolerance scores of the RIL population, the cold-sensitive parents also provided positive alleles at some loci. This agrees with the observation that the percent LTSS and percent EL range of the RIL population was wider than the range between the two AUS parents (see **Table 1**), thus explaining the observed “transgression” values when positive or negative alleles from each parent were combined in individual RILs. This is particularly noteworthy for the EL trait, where both parents had a mean of 25%, while the RILs had a range of 15%–54% (**Table 1**).

**Table 4:** Cold tolerance quantitative trait loci (QTL) for percent electrolyte leakage (EL, BLUP) and low-temperature survivability (LTSS, both a percent BLUP and logit) identified in the NC 1/536 × Phudugey *aus* (AUS) and Taichu Mochi 59 × British Honduras Creole *tropical japonica* (TRJ) recombinant inbred line (RIL) mapping populations.

QTL^a^	Chr.	QTL region (cM)^b^	QTL interval (Mb)^c^	Marker nearest LOD peak	Peak position (cM)	LOD value^d^	Additive effect^e^	PVE^f^	Gene symbol^g^	Position (Mb)^h^	LOC_ID^i^	Citation
NC 1/536 x Phudugey AUS RIL population												
*qEL8^(BLUP)^* ^j^	8	23.1–23.4	19.5–20.4	8_20111664	23.3	3.16	2.10	9.0	*Badh2*	20.39	LOC_Os08g32870	Chen et al. (2008)
									*OsANN7*	20.46	LOC_Os08g32970	Singh et al. (2014); Que et al. (2023)
									*OsFBDUF45*	20.48	LOC_Os08g33010	This study
*qEL10^(BLUP)^* ^j^	10	0.6–2.0	4.26–9.7	10_5714528	1.8	2.19	− 1.74	6.3	*OsWAK22*	4.08	LOC_Os10g07556	Brusamarello-Santos et al. (2019)
*qEL11^(BLUP)^* ^j^	11	34.4–35.0	26.5–27.6	11_26484157	34.4	2.61	1.89	7.5	*OsPRX134*	26.60	LOC_Os11g43980	This study
									*OsRLCK352*	27.57	LOC_Os11g45540	This study
									*OsU2AF65B*	27.59	LOC_Os11g45590	Meng et al. (2025)
*qLTSS1–1^(logit)^* ^j^	1	29.0–30.3	14.0 - 15.2	1_13988902	29.1	4.94	-0.42	13.7	*IPI1*	14.02	LOC_Os01g24880	Wang et al. (2017)
*qLTSS1–1^(percent)^* ^j^	1	28.7–30.3	13.8–15.8	1_13988902	29.0	4.66	− 5.65	13.0	Esterase	14.33	LOC_Os01g25360	This study
									*OsWAK6*	14.86	LOC_Os01g26210	Panahabadi et al. (2022)
									*OsC2DP4*	15.16	LOC_Os01g27190	This study
									*OsLAC1*	15.45	LOC_Os01g27700	Liu et al. (2017)
*qLTSS1–2^(logit)^*	1	52.2–56.1	40.5–41.5	1_41089199	53.4	2.79	0.32	8.0	*OsCAMTA5*	40.40	LOC_Os01g69910	Kim et al. (2014)
*qLTSS1–2^(percent)^*	1	52.1–56.4	40.5–41.7	1_40455716	53.2	3.18	4.81	9.1	*Hsp40* (DnaJ)	40.68	LOC_Os01g70250	This study
*qLTSS6^(logit)^*	6	0.0–2.6	2.0–3.4	6_2010737	0.9	13.18	− 0.77	32.6	*RLP32*	2.10	LOC_Os06g04810	This study
*qLTSS6^(percent)^*	6	0.0–2.6	2.0–3.4	6_2010737	1.2	13.56	− 11.04	33.3	*OsMKK1*	2.50	LOC_Os06g05520	Wang et al. (2014); Wu et al. (2025)
									*OsNAPL2*	2.56	LOC_Os06g05660	Tripathi et al. (2015); Schläppi et al. (2023)
									Ubiquitin	2.61	LOC_Os06g05760	Nischal et al. (2012)
									Transferase	2.63	LOC_Os06g05790	Schläppi et al. (2023)
									*RRM1-115*	2.64	LOC_Os06g05800	Jiang et al. (2024)
									Esterase	2.80	LOC_Os06g06080	Schläppi et al. (2023)
									*OsPUB64*	3.04	LOC_Os06g06470	Kim et al. (2021)
									*OsGELP80*	3.06	LOC_Os06g06520	Sun et al. (2022)
*qLTSS11^(logit)^* ^j^	11	33.5–34.8	25.4–27.0	11_26484157	34.4	10.49	− 0.64	26.9	See above *qEL11^(BLUP)^*^j^			
*qLTSS11^(percent)^* ^j^	11	34.4–35.0	26.5–27.6	11_26484157	34.7	10.22	− 8.92	26.3	See above *qEL11^(BLUP)^*^j^			
Taichu Mochi 59 × British Honduras Creole TRJ RIL population												
*qEL4^(BLUP)^* ^j^	4	37.0–39.8	30.5–31.4	4_31350359	38.4	3.08	2.88	14.3	*OsIRL1*	30.55	LOC_Os04g51580	You et al. (2010)
*qEL11^(BLUP)j^*	11	16.4–17.1	9.9–13.7	11_9864107	16.5	2.96	− 2.65	13.8				
*qLTSS4^(logit)^* ^j^	4	37.0–38.9	29.8–31.4	4_30483002	37.1	2.27	− 0.39	10.8	See above *qEL4^(BLUP)^*^j^			
*qLTSS7–1^(percent)^* ^j^	7	40.3–41.4	25.9–26.75	7_26640591	40.8	2.55	9.75	12.0	Expressed	26.19	LOC_Os07g43770	Jahan et al. (2019)
*qLTSS7–2^(logit)^* ^j^	7	42.3–44.6	28.0–29.0	7_28989771	44.7	2.37	− 0.39	11.2	*OsTBL24*	27.59	LOC_Os07g46230	This study
									*OsCHLH*	27.62	LOC_Os07g46310	Jung et al. (2003); Miyao et al. (2007)
									*OsANN6*	27.80	LOC_Os07g46550	Zhang et al. (2025)
									*OsUBP10*	27.89	LOC_Os07g46660	Zou et al. (2023)
									*OsPHD45*	27.90	LOC_Os07g46690	Sun et al. (2017)
*qLTSS10^(logit)^* ^j^	10	10.4–11.8	11.1–11.6	10_11051662	11.0	2.45	− 0.42	11.6	ATPase	11.79	LOC_Os10g22700	This study
									Expressed	11.84	LOC_Os10g22770	This study
									Cf2/Cf5	11.92	LOC_Os10g22930	Wang et al. (2018)
									*OsABH1*	11.95	LOC_Os10g22960	Jin et al. (2024)
									*OsbHLH045*	12.02	LOC_Os10g23050	Li et al. (2006); Palaniswamy et al. (2024)

Known candidate genes located in the QTL regions are listed. The AUS RIL population had 153 progeny genotyped with 516 SNP markers, and the TRJ RIL population had 90 progeny genotyped with 1,042 SNP markers.

a Quantitative trait loci (QTL) are declared using Qgene version 4.4.0 (Joehanes and Nelson, 2008) with single-trait multiple interval mapping and a default scan size of 10 millimorgans (mM). After initial QTL identification, the scan size was reduced to 1 mM, and QTL mapping was rerun to focus on the chromosomes containing the QTL regions.

b The QTL region is defined in centimorgans (cM) based on the linkage map.

c The QTL interval is defined by the megabase positions of the DNA markers closest to the QTL region boundaries.

d QTL were considered significant if the LOD value was about 3.5 (α = 0.05, 1,000 permutations). LOD values around 2.7 were considered significant at α = 0.10 (1,000 permutations).

e A positive additive effect indicates that the NC 1/536 or Taichu Mochi 59 allele increases the phenotype for that trait. A negative additive effect indicates that the Phudugey or British Honduras Creole allele increases the phenotype for the trait.

f PVE is the percentage of total phenotypic variation explained by an individual QTL, as estimated by *R*^2^ values in the Qgene analysis.

g Gene nomenclature follows the standardized rice gene nomenclature in *Oryzabase* (Yamazaki et al., 2010), using the Committee on Gene Symbolization, Nomenclature, and Linkage gene symbols.

h The physical position of the candidate gene is based on the Rice Genome Annotation Project Release 7.

i Rice Genome Annotation Project locus identifier for the candidate gene (Kawahara et al., 2013).

j Rice subpopulation-specific QTL not previously uncovered by TEJ × AUS mapping populations (Schläppi et al., 2023).

For *qLTSS* mapping in the TRJ subpopulation, increased seedling survivability associated with three of the four LTSS QTL—*qLTSS4*, *qLTSS7-2*, and *qLTSS10*—was attributed to alleles from the very cold-tolerant British Honduras Creole parent, while for *qEL* mapping, increased electrolyte leakage and thus increased membrane damage after cold exposure associated with *qEL4* and *qEL11* was attributed to alleles from the more cold-sensitive Taichu Mochi 59 parent and the more tolerant British Honduras Creole parent, respectively (**Table 4**). This again showed, as for the AUS subpopulation, that both TRJ parents contributed positive or negative alleles to enhance or reduce cold tolerance scores, respectively, because the percent LTSS and percent EL range of the RIL population was wider than the range between the two TRJ parents (**Table 2**).

### Identification of cold tolerance candidate genes

One rationale for the current study was to identify rice subpopulation-specific cold tolerance QTL and associated candidate genes that could be used to improve cold tolerance traits within the two rice subpopulations. Due to the lower density of SNPs within subpopulation RILs compared to RILs generated from crosses between parents from different subpopulations, the 13 cold tolerance trait QTL identified here covered larger genomic regions than those we mapped previously using TEJ × AUS biparental crosses (Schläppi et al., 2023). Interestingly, only two AUS QTL overlapped with our previously mapped QTL from TEJ × AUS biparental, *qLTSS1–2*^(logit and percent)^ and *qLTSS6*^(logit and percent)^, while none of the TRJ QTL did (**Table 4**; Schläppi et al., 2023). This showed that our approach indeed uncovered subpopulation-specific QTL and associated cold tolerance candidate genes. However, the larger QTL regions overlapped with several smaller QTL we previously identified using genome-wide association studies (**Supplementary Table S5**; Schläppi et al., 2017; Shimoyama et al., 2020).

To identify EL and LTSS trait candidate genes, we searched within the 13 QTL regions for mostly high-impact single-nucleotide polymorphisms (SNPs) or insertion/deletion (InDel) variants in genes leading to frameshift, premature termination, lost start codon, lost stop codon, or splice variants. Altogether, 23 and 12 genes with variants were identified for the AUS and TRJ RILs, respectively (**Table 5**). For the AUS QTL *qEL8*^(BLUP)^ and *qEL11*^(BLUP)^, alleles from the cold-sensitive parent increased EL and thus membrane damage after cold exposure. Consistently, high-impact variants were identified in alleles of the cold-sensitive parent for all seven candidate genes listed in **Table 4**. Specifically, biological functions for candidate genes encoding a peroxidase (*OsPRX134*), a receptor-like cytoplasmic kinase (*OsRLCK352*), and a potential splice regulator (*OsU2AF65B*) associated with *qEL11*^(BLUP)^, and genes coding for an aldolase (*Badh2*), annexin (*OsANN7*), and F-box factor (*OsFBDUF45*) associated with *qEL8*^(BLUP)^, were most likely disrupted in the cold-sensitive AUS parent. Among these candidate genes, *OsANN7* might be of particular interest because it was recently shown that another annexin family member, *OsANN5*, is involved in cold stress tolerance at the seedling stage (Que et al., 2023). In contrast, for the AUS QTL *qEL10*^(BLUP)^, alleles from the cold-tolerant parent increased EL, and consistently, a high-impact variant was identified in a wall-associated kinase (*OsWAK22*) of the cold-tolerant parent. Disruption of the biological function of this candidate gene might increase membrane damage after cold exposure, possibly because signal transduction from the cell wall across the plasma membrane to the cytoplasm might be partially compromised. Correct signaling by *OsWAK22* might dampen immune responses during cold temperature exposure (Brusamarello-Santos et al., 2019).

**Table 5:** High-impact SNP and InDel variants in *aus* parents, comparing the cold-sensitive NC 1/536 with cold-tolerant Phudugey, and in *tropical japonica* parents, comparing the cold-sensitive Taichu Mochi 59 with cold-tolerant British Honduras Creole.

Chr.	Gene annotation	RAPDB locus ID	MSU locus id	Variation id	Nucleotide change	Impact
1	*IPI1*	Os01g0350900	LOC_Os01g24880	vg0114019098	G>GGAGAA	Pro440fs
1	Esterase	Os01g0355800	LOC_Os01g25360	vg0114326854	A>ACGGCG	Lys203fs^a^
1	*OsWAK6*	Os01g0364400	LOC_Os01g26210	vg0114858164	TGC>T	Gln36fs
1	*OsC2DP4*	Os01g0369500	LOC_Os01g27190	vg0115161009	CCGCG>C	Ala197fs
1	*OsLAC1*	Os01g0374600	LOC_Os01g27700	vg0115446885	A>T	Lys336*
1	*OsCAMTA5*	Os01g0923600	LOC_Os01g69910	vg0140402807	G>A	Splice variant
1	*Hsp40*	Os01g0927400	LOC_Os01g70250	vg0140675241	A>G	Ter761Glnext*?^a^
4	*OsIRL1*	Os04g0605300	LOC_Os04g51580	vg0430553339	A>ATTCAGCCTCTCAG	Ter353fs
6	*RLP32*	Os06g0140000	LOC_Os06g04810	vg0602096493	AT>A	Ile774fs
6	*OsMKK1*	Os06g0147800	LOC_Os06g05520	vg0602499219	T>A	Ter353Leuext*?
6	*OsNAPL2*	Os06g0149400	LOC_Os06g05660	vg0602560534	G>T	Splice variant^a^
6	Ubiquitin	Os06g0150800	LOC_Os06g05760	vg0602608137	G>A	Trp86*
6	Transferase	Os06g0151100	LOC_Os06g05790	vg0602633368	TC>T	Glu515fs^a^
6	*RRM1–115*	Os06g0151200	LOC_Os06g05800	vg0602639176	CCATTACCAT>C	Splice variant, Asp161del
6	Esterase	Os06g0154400	LOC_Os06g06080	vg0602803109	C>CGA	Leu24fs
6	*OSPUB64*	Os06g0159600	LOC_Os06g06470	vg0603043771	A>T	Lys197*
6	*OsGELP80*	Os06g0160200	LOC_Os06g06520	vg0603058928	G>GCCGC	Leu14fs
7	Expressed	Os07g0631300	LOC_Os07g43770	vg0726188889	CGAGAGA>CGAGAGAGAGA	Cys82fs^a^
7	*OsTBL24*	Os07g0656000	LOC_Os07g46230	vg0727587071	GAC>G	Thr346fs
7	*OsCHLH*	Os07g0656500	LOC_Os07g46310	vg0727624302	CTCA>C	Tyr721fs
7	*OsANN6*	Os07g0659600	LOC_Os07g46550	vg0727800858	C>G	Splice variant^a^
7	*OsUBP10*	Os07g0661300	LOC_Os07g46660	vg0727887682	G>T	Splice variant
7	*OsPHD45*	Os07g0661500	LOC_Os07g46690	vg0727904649	G>A	Arg1601*
8	*Badh2*	Os08g0424500	LOC_Os08g32870	vg0820385593	T>TG	Trp459fs
8	*OsANN7*	Os08g0425700	LOC_Os08g32970	vg0820461233	A>C	Tyr284Ser
8	*OsFBDUF45*	Os08g0426100	LOC_Os08g33010	vg0820480872	C>A	Gly219*
10	*OsWAK22*	Os10g0162844	LOC_Os10g07556	vg1004081143	G>T	Ser160*^a^
10	ATPase	None	LOC_Os10g22700	vg1011792594	TG>T	Leu115fs
10	Expressed	None	LOC_Os10g22770	vg1011836758	T>A	Lys181*
10	Cf2/Cf5	Os10g0375400	LOC_Os10g22930	vg1011914935	AGGGG>A	Gly92fs
10	*OsABH1*	Os10g0375700	LOC_Os10g22960	vg1011947270	GCC>G	Ala12fs
10	*OsbHLH045*	Os10g0376900	LOC_Os10g23050	vg1012016133	C>CCGCCGG	Ala36_Gly37insAlaGly
11	*OsPRX134*	Os11g0661600	LOC_Os11g43980	vg1126573539	C>CGG	Leu227fs
11	*OsRLCK352*	Os11g0681400	LOC_Os11g45540	vg1127573677	C>CAT	Gly146fs
11	*OsU2AF65B*	Os11g0682300	LOC_Os11g45590	vg1127592284	AAGGGACAGGGAT>AGGGATAGGGACAGGGAT	Arg72fs

ext*?, stop codon lost, reading frame extended; *fs*, frame shift; *, premature stop codon.

a Nipponbare reference matches the less cold-tolerant variety (Taichu Mochi 59 or NC_1/536).

For AUS QTL *qLTSS1–1*^(logit and percent)^, *qLTSS6*^(logit and percent)^, and *qLTSS11*^(logit and percent)^, alleles from the cold-tolerant parent increased the low-temperature seedling survival trait. Consistently, high-impact variants were identified in alleles of the cold-sensitive parent for most of these QTL candidate genes listed in **Table 4**, including *qLTSS11*^(logit and percent)^, which overlaps with *qEL11*^(BLUP)^ (see above). Thus, defects in the biological functions of at least one of *qLTSS1–1*^(logit and percent)^-associated candidate genes coding for *IPA1 interacting protein 1* (*IPI1*), *OsWAK6*, a C2 calcium-dependent membrane targeting domain containing protein (*OsC2DP4*), or *Laccase 1* (*OsLAC1*) might have negative effects on cold tolerance. Interestingly, the cold-tolerant AUS parent has a frameshift in an esterase-encoding gene (LOC_Os01g25360), which may have no consequence or could even positively affect cold tolerance. Moreover, in the cold-sensitive parent, defects in the biological functions of at least one of *qLTSS6*^(logit and percent)^-associated candidate genes—coding for a resistance protein (*RLP32*), *MAP kinase kinase 1* (*OsMKK1*; described in Schläppi et al., 2017), a ubiquitin family gene (LOC_Os06g05760), an RNA-binding protein (*RRM1–115*), an esterase (LOC_Os06g06080; described in Schläppi et al., 2023), a U-box E3-ubiquitin ligase (*OsPUB64*), or a Gly-Asp-Ser-Leu (GDSL)-type esterase/lipase (*OsGELP80*)—might negatively affect cold tolerance. Of special interest is *OsMKK1*, which we previously flagged as a candidate gene in Schläppi et al. (2017) and which is downregulated by cold temperatures (**Table 6**), possibly to dampen cold stress signaling events. Of further interest are *IPI1*, LOC_Os06g05760, and *OsPUB64*, because, first, they belong to ubiquitin and E3-ubiquitin ligase family genes that might be involved in regulating protein homeostasis in response to cold temperature stress (Wang et al., 2017; Kim et al., 2021); second, *IPI1* and *OsPUB64* are upregulated by cold temperatures (**Table 6**); and third, we recently have shown that *OsUBC7*, an E2-ubiquitin conjugase-encoding gene, is involved in the early cold tolerance response of rice (Phan and Schläppi, 2025). Thus, *IPI1* and *OsPUB64* might belong to a similar biochemical pathway as previously identified rice cold tolerance genes, such as *OsUBC7*, *OsHOS1*, and *OsSRFP1*—two E3-ubiquitin ligase-encoding genes (Lourenço et al., 2013; Fang et al., 2015). Interestingly, the cold-tolerant AUS parent also contributed defective alleles of genes coding for a histone chaperone (*OsNAPL2*; described in Schläppi et al., 2023) and a transferase (LOC_Os06g05790; described in Schläppi et al., 2023), which either had no effect or a positive effect on cold tolerance. By contrast, for AUS QTL *qLTSS1-2*^(logit and percent)^, alleles from the cold-sensitive parent increased low-temperature seedling survival. Consistently, the cold-tolerant parent had a high-impact variant in a gene coding for a heat-shock chaperone DnaJ (*Hsp40*), while the cold-sensitive parent had the (most likely) functional reference allele. However, the second candidate gene, coding for a calmodulin-binding transcription activator (*OsCAMTA5*), had a splice acceptor variant (vg0140402807) in the cold-sensitive parent, which either has no effect or might positively affect calcium signaling during cold stress, meriting further investigation.

**Table 6:** Cold temperature-regulated gene expression of QTL-associated candidate genes with high-impact variants between cold-tolerant and cold-sensitive rice accessions.

Chr.	Gene annotation	RAPDB locus id	MSU locus id	Fold change (PRJNA610422)^b^	Fold change (PRJNA430015)^c^
1	*IPI1*	Os01g0350900	LOC_Os01g24880	− 1.36	1.34
1	Esterase	Os01g0355800	LOC_Os01g25360	ns	− 3.22
1	*OsWAK6*	Os01g0364400	LOC_Os01g26210	ns	− 4
1	*OsC2DP4*	Os01g0369500	LOC_Os01g27190	ns	ns
1	*OsLAC1*	Os01g0374600	LOC_Os01g27700	ns	ns
1	*OsCAMTA5*	Os01g0923600	LOC_Os01g69910	− 1.45	− 2.04
1	*Hsp40*	Os01g0927400	LOC_Os01g70250	− 1.23	−3.59
4	*OsIRL1*	Os04g0605300	LOC_Os04g51580	ns	ns
6	*RLP32*	Os06g0140000	LOC_Os06g04810	2.1	ns
6	*OsMKK1*	Os06g0147800	LOC_Os06g05520	− 1.15	ns
6	*OsNAPL2*	Os06g0149400	LOC_Os06g05660	ns	− 1.66
6	Ubiquitin	Os06g0150800	LOC_Os06g05760	na	na
6	Transferase	Os06g0151100	LOC_Os06g05790	− 1.55	− 1.57
6	*RRM1–115*	Os06g0151200	LOC_Os06g05800	− 1.42	ns
6	Esterase	Os06g0154400	LOC_Os06g06080	− 1.34	ns
6	*OSPUB64*	Os06g0159600	LOC_Os06g06470	1.5	ns
6	*OsGELP80*	Os06g0160200	LOC_Os06g06520	ns	ns
7	Expressed	Os07g0631300	LOC_Os07g43770	ns	1.61
7	*OsTBL24*	Os07g0656000	LOC_Os07g46230	5.7	ns
7	*OsCHLH*	Os07g0656500	LOC_Os07g46310	− 1.65	ns
7	*OsANN6*	Os07g0659600	LOC_Os07g46550	ns	ns
7	*OsUBP10*	Os07g0661300	LOC_Os07g46660	1.2	ns
7	*OsPHD45*	Os07g0661500	LOC_Os07g46690	1.3	ns
8	*Badh2*	Os08g0424500	LOC_Os08g32870	1.1	ns
8	*OsANN7*	Os08g0425700	LOC_Os08g32970	ns	ns
8	*OsFBDUF45*	Os08g0426100	LOC_Os08g33010	ns	ns
10	*OsWAK22*	Os10g0162844	LOC_Os10g07556	ns	ns
10	ATPase	None	LOC_Os10g22700	ns	na
10	Expressed	None	LOC_Os10g22770	na	na
10	Cf2/Cf5	Os10g0375400	LOC_Os10g22930	ns	ns
10	*OsABH1*	Os10g0375700	LOC_Os10g22960	− 1.33	ns
10	*OsbHLH045*	Os10g0376900	LOC_Os10g23050	− 3.44	ns
11	*OsPRX134*	Os11g0661600	LOC_Os11g43980	ns	ns
11	*OsRLCK352*	Os11g0681400	LOC_Os11g45540	− 15.29	ns
11	*OsU2AF65B*	Os11g0682300	LOC_Os11g45590	ns	ns

*ns*, no significant change; *na*, no available expression data.

a The data were obtained from Yu et al. (2022).

b NCBI Project ID PRJNA610422: “4°–0 h” used as the warm control and “4°–12 h” as the cold-treated sample.

c NCBI Project ID PRJNA430015: “CTR control” used as the warm control and “CTR cold stress” as the cold-treated sample. Additional details are reported in **Supplementary Table S6**.

Taken together, the AUS subpopulation-specific QTL mapping for the EL and LTSS traits provided 23 candidate genes involved in phospholipid binding, lipase activity, calcium and kinase signaling, immune response, RNA binding, and ubiquitin-mediated protein turnover. Most of these candidate genes likely carried functional reference alleles from the cold-tolerant parent, correlating with enhanced cold tolerance potential. An additional interesting candidate gene is *Badh2*, which encodes a betaine aldehyde dehydrogenase that inhibits the biosynthesis of the major rice fragrance component 2-acetyl-1-pyrroline (Chen et al., 2008) and is upregulated by cold (**Table 6**). Whether variants of this gene for eating quality also influence cold tolerance merits further investigation; however, it is noteworthy that its paralog, *Badh1*, plays a positive role in rice cold stress tolerance (Tang et al., 2014).

For overlapping TRJ QTL *qEL4*^(BLUP)^ and *qLTSS4*^(logit)^, alleles from the relatively cold-sensitive parent increased EL and decreased LTSS, respectively. Only one candidate gene, coding for an intracellular Ras-group-related leucine-rich repeat (LRR) family protein (*OsIRL1*), was identified, which had a frameshift variant allele in the relatively cold-sensitive parent. This defect might negatively affect stress tolerance signaling during cold exposure (You et al., 2010) and merits further investigation. For TRJ QTL *qEL11*^(BLUP)^, alleles from the cold-tolerant parent increased EL; however, no candidate gene with high-impact variants was identified. For TRJ QTL *qLTSS7-2*^(logit)^ and *qLTSS10*^(logit)^, alleles from the cold-tolerant parent increased LTSS. Consistently, almost all candidate genes identified in the QTL regions carried high-impact variants in alleles from the relatively cold-sensitive parent. Defects in genes coding for a trichome birefringence-like protein (*OsTBL24*), a magnesium chelatase (*OsCHLH*), a ubiquitin carboxyl-terminal hydrolase domain containing protein (*OsUBP10*), and a PHD-finger family protein (*OsPHD45*), associated with *qLTSS7-2*^(logit)^, as well as genes coding for an AAA-type ATPase (LOC_Os10g22700), a hypothetical protein (LOC_Os10g22770), a Cf2/Cf5 disease resistance protein (LOC_Os10g22930), a triacylglycerol lipase (*OsABH1*), and a basic helix–loop–helix factor (*OsbHLH045*), associated with *qLTSS10*^(logit)^, might have negative effects on cold tolerance. Only one gene associated with *qLTSS7-2*^(logit)^, *OsANN6*, had a high-impact splice donor variant in the cold-tolerant parent, which either had no effect or positively affected cold tolerance. In contrast, for TRJ QTL *qLTSS7-1*^(logit)^, alleles from the relatively cold-sensitive parent increased LTSS. Only one candidate gene coding for a hypothetical protein (LOC_Os07g43770) was identified, which had a high-impact variant in the cold-tolerant parent, indicating that the reference allele might have a positive effect on cold tolerance.

In addition to *OsIRL1*, additional TRJ genes of interest that might merit further investigation include the following: *OsCHLH*, for which transposon insertions are lethal due to an albino phenotype (Jung et al., 2003; Miyao et al., 2007), whereas the variant uncovered here (**Table 5**) is viable and downregulated by cold (**Table 6**); *OsPHD45*, which might regulate growth and development during cold stress and is upregulated by cold (**Table 6**); LOC_Os10g22930, previously identified as a candidate gene for low-temperature tolerance during germination (Wang et al., 2018); and the transcription factor encoding gene *OsbHLH045*, expressed in the root (Li et al., 2006), downregulated by cold (**Table 6**), and potentially regulating cold stress-responsive genes in this organ, as well as possibly playing a role in carotenoid biosynthesis (Palaniswamy et al., 2024). Finally, *OsABH1* is another interesting candidate because this lipase affects cellular triglyceride content, possibly influences cell wall components, and is downregulated by cold, which might positively affect cold tolerance potential (Jin et al., 2024).

## Conclusions

In this study, we used AUS and TRJ subpopulation-specific biparental mapping populations, derived from crosses between cold-tolerant and cold-sensitive parents, to map subpopulation-specific cold tolerance QTL. We first performed “quality control” QTL analyses to demonstrate that QTL for heading date, plant height, and culm habit contained known genes/QTL for all traits, such as *MADS50*, *RFT1*, *HD2*, *PK1*, *GA2OX3*, *CSN1*, *SDF5*, *OsGDI1*, *TAD1*, and *WFP*, thus validating that the populations were suitable for cold tolerance trait QTL discovery (**Table 3**). Only two of the 13 cold tolerance QTL identified here overlapped with the QTL we previously obtained through biparental mapping using different subpopulation parents, indicating that this approach identified subpopulation-specific QTL that could be used to improve the cold tolerance potential of each respective subpopulation. We identified 35 candidate genes for cold tolerance in the QTL regions—23 for the AUS subpopulation and 12 for the TRJ subpopulation—and 80% (28/35) had gene-disruptive nucleotide variants in the cold-sensitive parent (**Table 5**). Based on allelic contributions to the QTL effect, we infer that the relatively cold-sensitive AUS and TRJ parents also contain functional genes that could be introduced into the cold-tolerant parents to further enhance their cold tolerance potential. The 35 candidate genes could be assigned to modules that regulate different cellular processes. Specifically, 10 genes (29%) encoded receptor-like (*OsRLCK*), wall-associated (*OsWAK*), MAPK, or other kinases, as well as ROS or calcium signaling components that, together with the three (9%) transcription factor genes *OsPHD45*, *OsbHLH045*, and *OsCAMTA5*, might be involved in transducing cold stress tolerance responses. Among the other 25 genes, five (14%) coded for ubiquitin- and F-box-type proteins, such as OsUBP10, IPA1, OsPUB64, and OsFBDUF45, which, together with the two (9% of AUS candidates) chaperone genes *OsNAPL2* and *Hsp40*, might regulate protein integrity and/or protein homeostasis as part of the cold stress tolerance response. Seven (20%) candidate genes coded for enzymes such as aldolases, esterases, laccases, lipases, and transferases that, together with the two (6%) RNA-binding genes *OsU2AF65B* and *RRM1–115*, might regulate cellular and RNA metabolic processes as part of specific cold stress tolerance response mechanisms. These candidate genes can be functionally analyzed in future studies using genomics, molecular genetics, and biochemical approaches, while the QTL regions containing them can be used for marker-assisted breeding of cold-tolerant rice cultivars.

## Data Availability

The original contributions presented in the study are included in the article/**Supplementary Material**. Further inquiries can be directed to the corresponding authors.

## Supplementary material

Supplementary Figure 1 Genetic distance between the AUS and TRJ recombinant inbred line parents using SSR genotypes of RMC accessions.

Supplementary Figure 2 **(A)** Images of NC 1/536 (GSOR 310381) and Phudugey (GSOR 310715) parents of the AUS recombinant inbred line (RIL) population. **(B)** Images of Taichu Mochi 59 (GSOR 310080) and British Honduras Creole (GSOR 310144), parents of the TRJ recombinant inbred line population. The eight TRJ RILs illustrate the diversity in the population.

Supplementary Figure 3 **(A)** Frequency distributions of 152 NC 1/536 x Phudugey AUS RILs evaluated for three seedling cold tolerance traits **(A)** percentage electrolyte leakage (EL), **(B)** low temperature seedling survivability-logit (LTSS logit) and **(C)** percentage LTSS (LTSS) and 153 RILs evaluated for three agronomic traits **(D)** days to 50% heading, **(E)** plant height and **(F)** culm habit, in the field near Stuttgart, Arkansas, USA. The Tukey outlier box plot illustrates the distribution for the traits evaluated. The diamond in the box indicates the mean, the line in the box indicates the median, and the whisker indicates the SE. The red bracket defines the shortest half of the data (the densest region). **(B)** Frequency distributions of 90 Taichu Mochi 59 (TM59) x British Honduras Creole (BHC) TRJ RILs evaluated for three seedling cold tolerance traits **(A)** percentage electrolyte leakage (EL), **(B)** low temperature seedling survivability logit (LTSS logit) and **(C)** percentage LTSS (LTSS) and 122 RILs evaluated for three agronomic traits **(D)** days to 50% heading, **(E)** plant height and **(F)** culm habit, in the field near Stuttgart, Arkansas, USA. The Tukey outlier box plot illustrates the distribution for the traits evaluated. The diamond in the box indicates the mean, the line in the box indicates the median, and the whisker indicates the SE. The red bracket defines the shortest half of the data (the densest region).

Supplementary Figure 4 **(A)** Correlation table and scatterplot matrix of the correlations between 153 NC 1/536 x Phudugey AUS RILs evaluated for three agronomic traits **(A)** days to 50% heading (HD), **(B)** plant height (PTHT) and **(C)** culm habit (CULMHAB) in the field near Stuttgart, Arkansas, USA and the 152 RILs evaluated for three seedling cold tolerance traits **(D)** percentage electrolyte leakage (EL), **(E)** percentage low temperature seedling survivability-blup (LTSS-blup) and **(F)** LTSS-logit (LTSS-logit). Correlations highlighted in blue are significant at *p* = 0.01 and in orange at *p*=<0.0001. Correlation coefficients were calculated using the multivariate option, restricted maximum likelihood (REML) method. **(B)** Correlation table and scatterplot matrix of the correlations between 122 Taichu Mochi 59 x British Honduras Creole TRJ RILs evaluated for three agronomic traits **(A)** days to 50% heading (HD), **(B)** plant height (PTHT) and **(C)** culm habit (CULMHAB) in the field near Stuttgart, Arkansas, USA and the 90 RILs evaluated for three seedling cold tolerance traits **(D)** percentage electrolyte leakage (EL), **(E)** percentage low temperature seedling survivability-blup (LTSS-blup) and F) LTSS-logit (LTSS-logit). Correlations highlighted in blue are significant at *p* = 0.01 and in orange at *p*=<0.0001. Correlation coefficients were calculated using the multivariate option, restricted maximum likelihood (REML) method.

Supplementary Table 1 List of the days to 50% heading (HD), plant height (PTHT) and culm habit (CULMHAB) for each AUS recombinant inbred line (RIL) and means for the parents, NC 1/536 and Phudugey. For the cold tolerance traits, the “adjusted means” were calculated for percent electrolyte leakage (EL) and low-temperature seedling survivability (LTSS), both BLUP and logit values are listed for each AUS RIL and the parents.

Supplementary Table 2 List of the days to 50% heading (HD), plant height (PTHT) and culm habit (CULMHAB) for each TRJ recombinant inbred line (RIL) and means for the parents, Taichu Mochi 59 (TM59) x British Honduras Creole (BHC). For the cold tolerance traits, the “adjusted means” were calculated for percent electrolyte leakage (EL) and low-temperature seedling survivability (LTSS), both BLUP and logit values are listed for each TRJ RIL and the parents.

Supplementary Table 3 List of the 516 single nucleotide polymorphism (SNP) markers from the Cornell-IR LD Rice Array (C7AIR) (Morales et al., 2020) that were polymorphic between the NC 1/536 and Phudugey, the AUS parents.

Supplementary Table 4 List of the 1,042 single nucleotide polymorphism (SNP) markers from the Cornell-IR LD Rice Array (C7AIR) (Morales et al., 2020) that were polymorphic between Taichu Mochi 59 and British Honduras Creole, the TRJ parents.

Supplementary Table 5 Overlap between AUS or TRJ QTL intervals listed on the left side and GWAS-QTL intervals identified in the Rice Mini-core Collection (RMC), Rice Diversity Panel 1 (RDP1), and the backcross inbred line (BIL) population which are listed on the right side. The GWAS-QTL found in the complete panel are designated as “ALL” and those found in a subgroup are designated as the panel and the subgroup, *Japonica* VG (JAP), *Indica* VG (IND) or a subpopulation, *tropical japonica* (TRJ) or *aus* (AUS).

Supplementary Table 6 Cold temperature regulation of AUS and TRJ RIL QTL associated candidate genes. The 22 candidate genes are highlighted in yellow and the expression data reported by Yu et al. (2022) is listed below the gene and highlighted in green. The significant difference was confirmed by *t*-tests highlighted in green.
