# Supplementary material for: Divergent genetic architecture of cold stress tolerance in aus and tropical japonica rice
Source: Front Plant Sci. 2026 Jan 7;16:1716845. doi: 10.3389/fpls.2025.1716845 (PMC12819804; doi:10.3389/fpls.2025.1716845)
Supplement: Supplementary Figure 1 — Genetic distance between the AUS and TRJ recombinant inbred line parents using SSR genotypes of RMC accessions. [file DataSheet1.pdf]

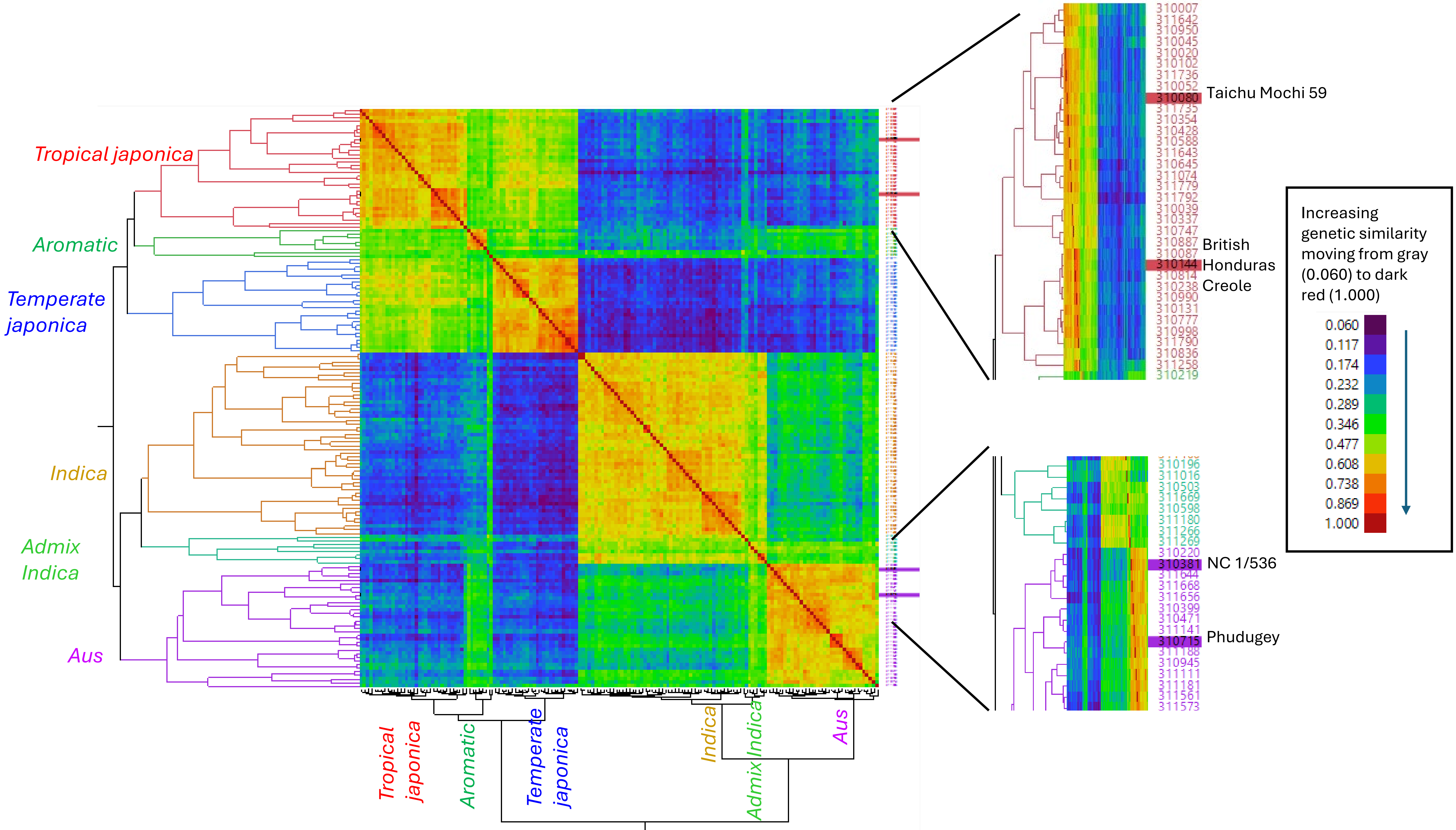

Tropical japonica
Aromatic
Temperate japonica
Indica
Admix Indica
Aus
310080 Taichu Mochi 59
310144 British Honduras Creole
310381 NC 1/536
310715 Phudugey
Increasing genetic similarity moving from gray (0.060) to dark red (1.000)
0.060
0.117
0.174
0.232
0.289
0.346
0.477
0.608
0.738
0.869
1.000

SUPPLEMENTARY FIGURE S1 | The genetic distance between 159 *O. sativa* accessions from the Rice Minicore (RMC) collection which were previously evaluated for cold tolerance at the seedling stage (Schlappi et al., 2017). The five major *O. sativa* subpopulations (*aus*, *indica*, *aromatic*, *temperate japonica* and *tropical japonica*) are identified on the left and bottom, in addition to admixture of *indica* cluster. These accessions were genotyped with 148 SSR markers, three InDel markers and six SNP markers. The distance matrix was generated using the Fast Ward method in JMP Genomics 12. The heat map and dendrogram show the genetic distance between accessions based on the distance matrix with similarities increasing as the value approaches 1.000 (red to dark red). The more distant accessions are dark blue to black (0.060). Based on the genetic distance and seedling cold tolerance rating two *aus* accessions, NC1/536 (GSOR 310381), which is cold susceptible and Phudugey (GSOR 310725), which is cold tolerant, both highlighted in purple, were selected to develop an *aus* RIL population. Similarly, two *tropical japonica* (TRJ) accessions Taichu Mochi 51(GSOR 310080) which is cold susceptible, and British Honduras Creole (GSOR 310144) which is cold tolerant, both highlighted in red, were selected to develop a recombinant inbred line (RIL) population in the TRJ background.

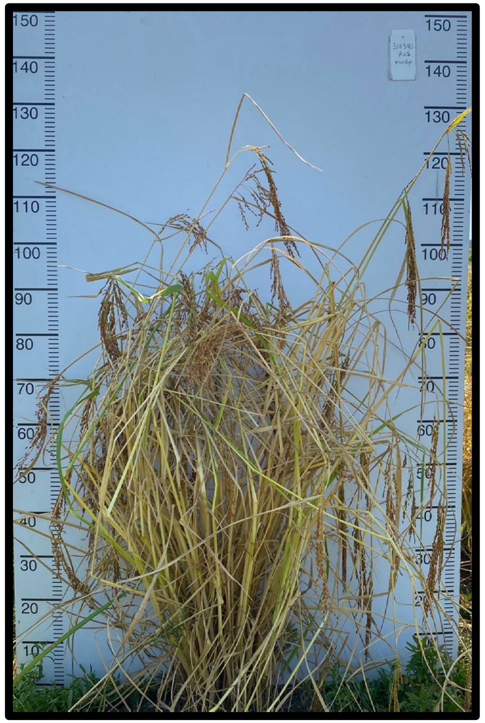

NC 1/536
GSOR 310381
Pakistan

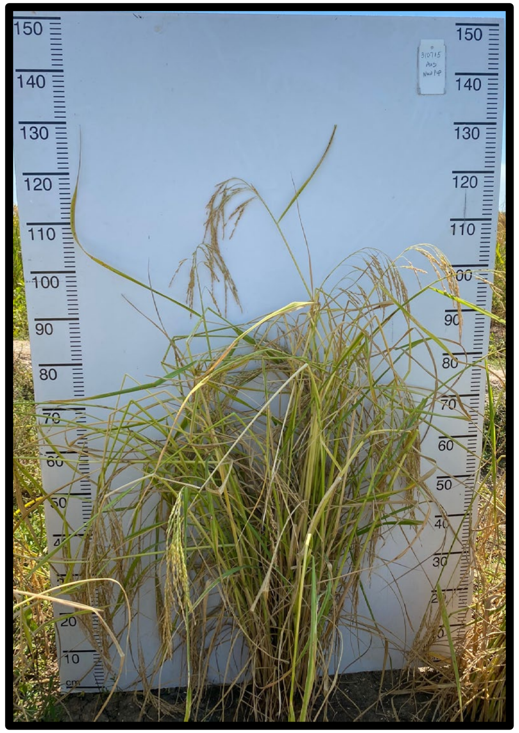

Phudugey
GSOR 310715
Bhutan

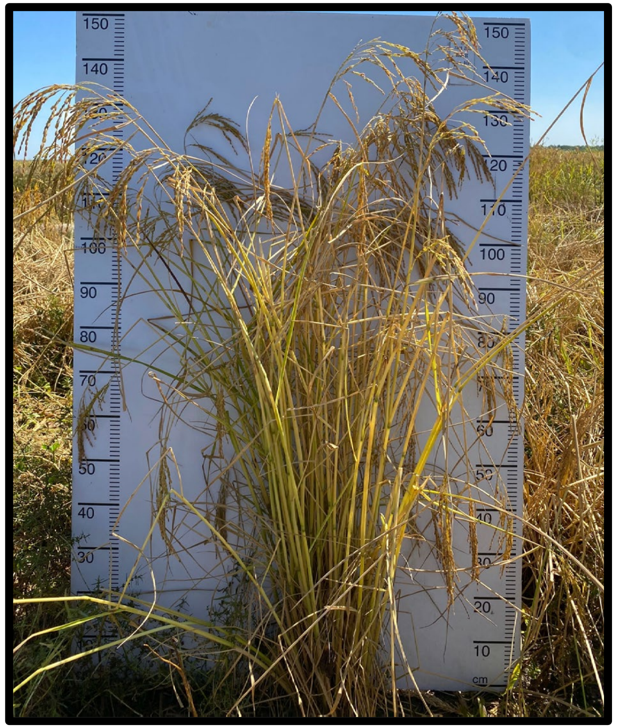

AUS RIL
aus_45

SUPPLEMENTARY FIGURE S2A | Images of NC 1/536 (GSOR 310381) from Pakistan and Phudugey (GSOR 310715) from Bhutan, parents of the *aus* (AUS) recombinant inbred line (RIL) population. The parents were selected from the Rice Minicore collection and classified as in the *aus* subpopulation. RIL aus_45 was extremely tall. Most of the AUS RILs lodged so it was difficult to get images. Images were from the field near Stuttgart, Arkansas, USA.

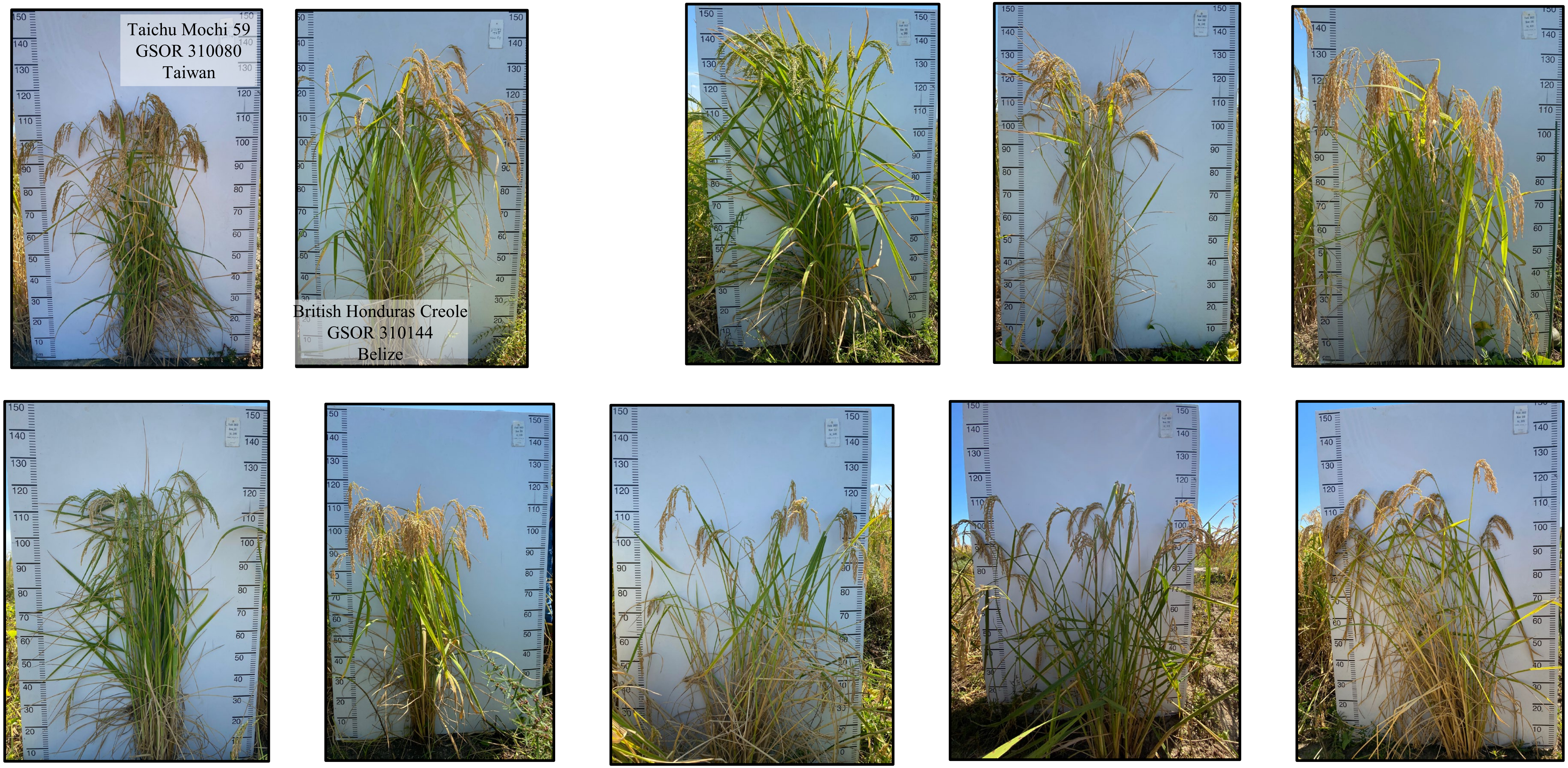

SUPPLEMENTARY FIGURE S2B | Images of Taichu Mochi 59 (GSOR 310080) and British Honduras Creole (GSOR 310144) in the upper left, parents of the *tropical japonica* (TRJ) recombinant inbred line (RIL) population. The parents were selected from the Rice Minicore collection and classified as in the TRJ subpopulation. The eight TRJ RILs illustrate the diversity for plant height and culm habit in the population. Images were from the field near Stuttgart, Arkansas, USA.

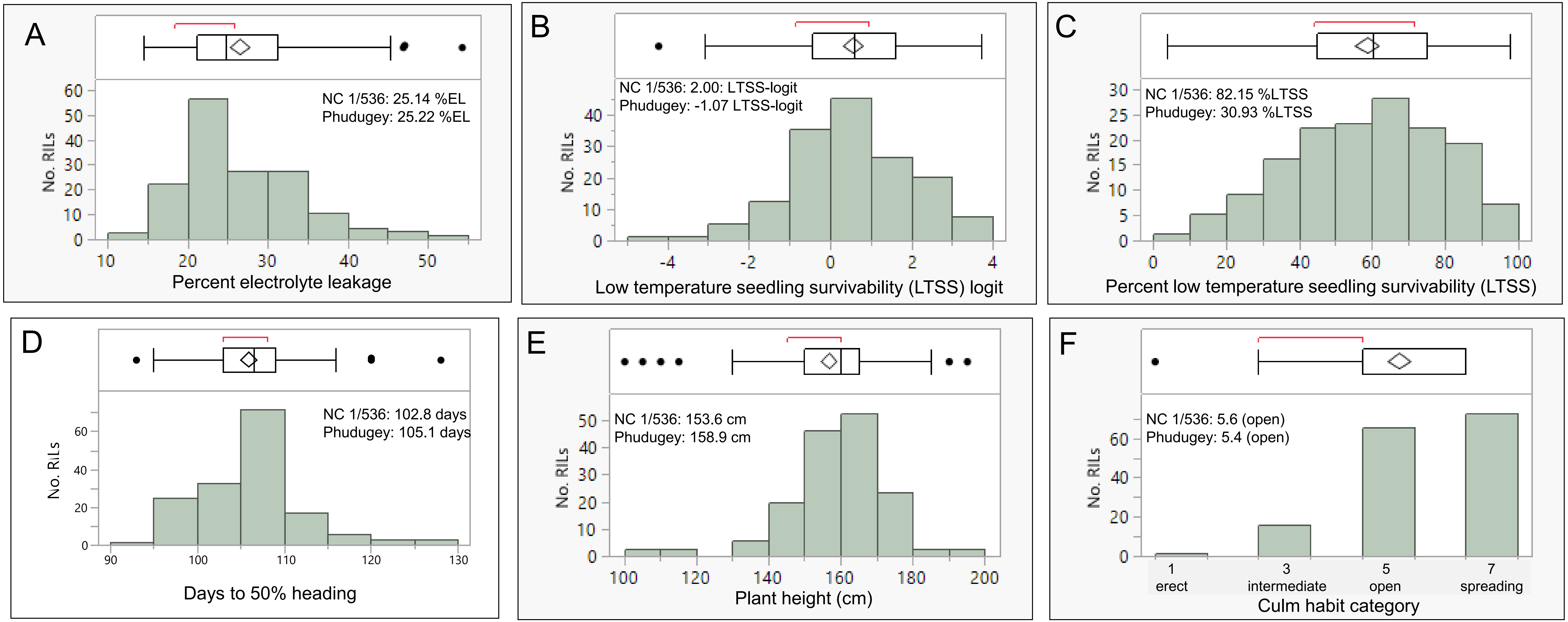

SUPPLEMENTARY FIGURE S3A | Frequency distributions of 152 NC 1/536 x Phudugey AUS RILs evaluated for three seedling cold tolerance traits A) percentage electrolyte leakage (EL), B) low temperature seedling survivability-logit (LTSS logit) and C) percentage LTSS (LTSS) and 153 RILs evaluated for three agronomic traits D) days to 50% heading, E) plant height and F) culm habit, in the field near Stuttgart, Arkansas, USA. The Tukey outlier box plot illustrates the distribution for the traits evaluated. The diamond in the box indicates the mean, the line in the box indicates the median, and the whisker indicates the SE. The red bracket defines the shortest half of the data (the densest region).

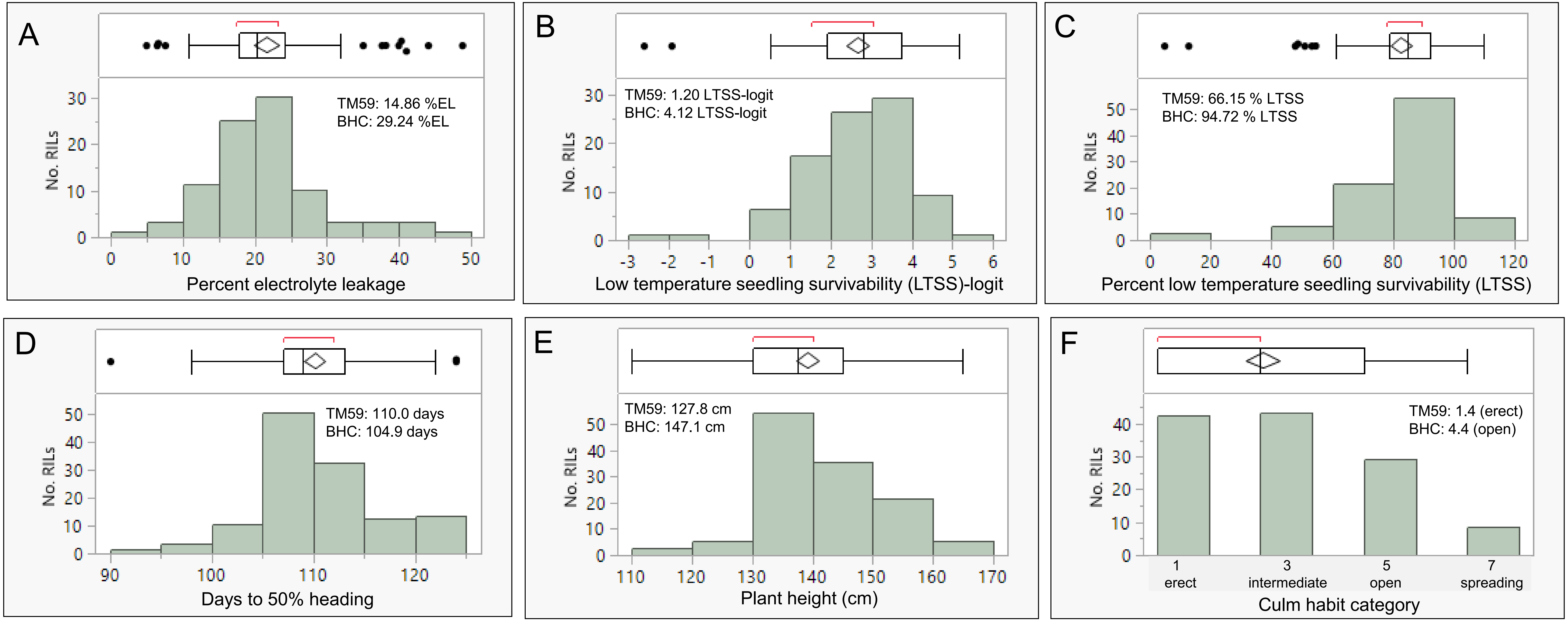

SUPPLEMENTARY FIGURE S3B | Frequency distributions of 90 Taichu Mochi 59 (TM59) x British Honduras Creole (BHC) TRJ RILs evaluated for three seedling cold tolerance traits A) percentage electrolyte leakage (EL), B) low temperature seedling survivability logit (LTSS logit) and C) percentage LTSS (LTSS) and 122 RILs evaluated for three agronomic traits D) days to 50% heading, E) plant height and F) culm habit, in the field near Stuttgart, Arkansas, USA. The Tukey outlier box plot illustrates the distribution for the traits evaluated. The diamond in the box indicates the mean, the line in the box indicates the median, and the whisker indicates the SE. The red bracket defines the shortest half of the data (the densest region).

Row	HD	PTHT	CULMHAB	EL	LTSS blup
PTHT	-0.32				
CULMHAB	-0.22	0.15			
EL	-0.05	0.04	0.04		
LTSS blup	-0.08	0.23	-0.02	-0.28	
LTSS logit	-0.12	0.26	-0.02	-0.26	0.98

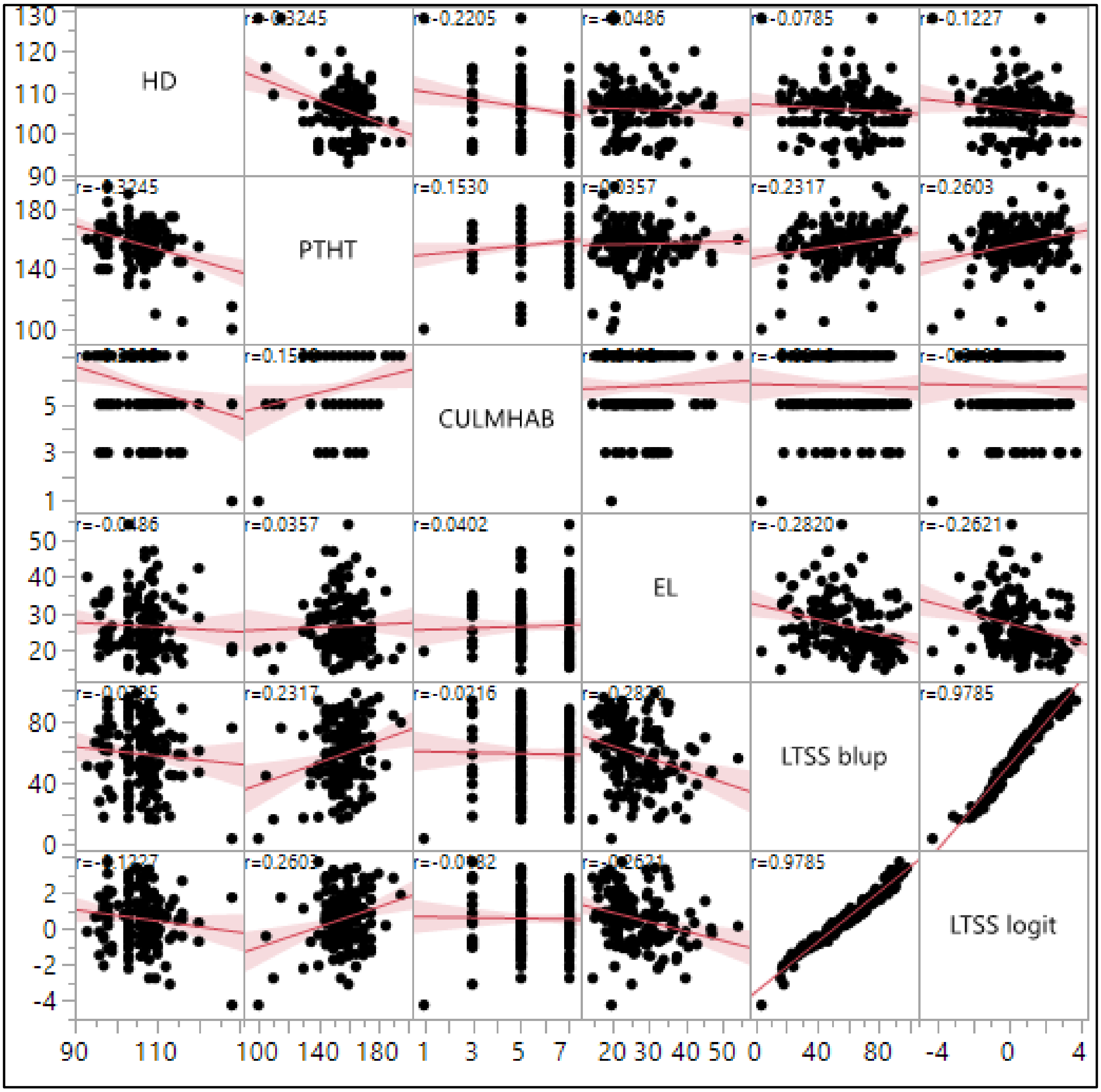

SUPPLEMENTARY FIGURE S4A | Correlation table and scatterplot matrix of the correlations between 153 NC 1/536 x Phudugey AUS RILs evaluated for three agronomic traits A) days to 50% heading (HD), B) plant height (PTHT) and C) culm habit (CULMHAB) in the field near Stuttgart, Arkansas, USA and the 152 RILs evaluated for three seedling cold tolerance traits D) percentage electrolyte leakage (EL), E) percentage low temperature seedling survivability-blup (LTSS-blup) and F) LTSS-logit (LTSS-logit). Correlations highlighted in blue are significant at *p*=0.01 and in orange at *p*=<0.0001. Correlation coefficients were calculated using the multivariate option, restricted maximum likelihood (REML) method.

Traits	HD	PTHT	CULMHAB	EL	LTSS blup
PTHT	0.06				
CULMHAB	-0.48	0.01			
EL	-0.06	-0.13	0.07		
LTSS blup	0.19	0.15	-0.28	-0.15	
LTSS logit	0.19	0.19	-0.30	-0.12	0.92

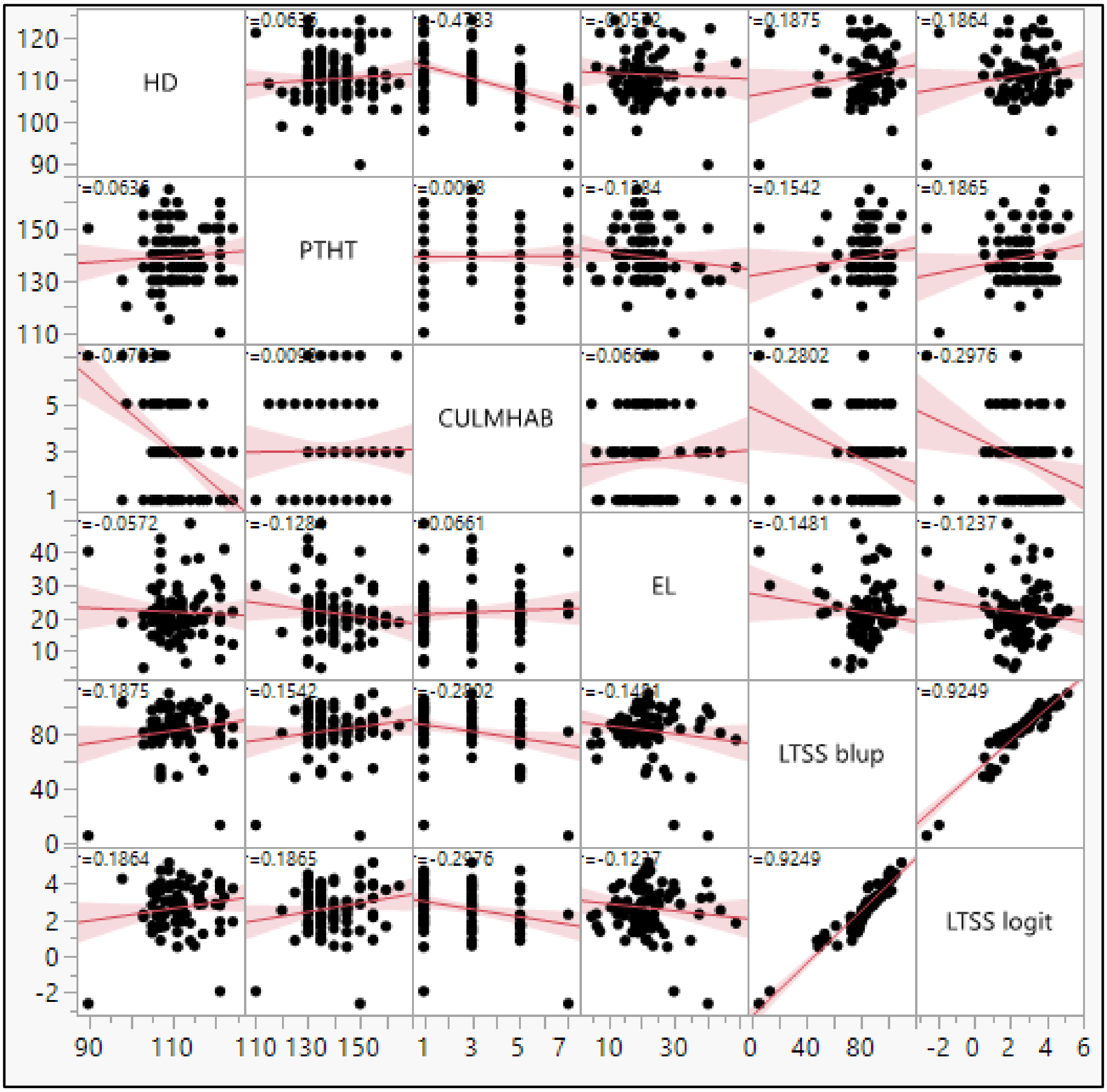

SUPPLEMENTARY FIGURE S4B | Correlation table and scatterplot matrix of the correlations between 122 Taichu Mochi 59 x British Honduras Creole TRJ RILs evaluated for three agronomic traits A) days to 50% heading (HD), B) plant height (PTHT) and C) culm habit (CULMHAB) in the field near Stuttgart, Arkansas, USA and the 90 RILs evaluated for three seedling cold tolerance traits D) percentage electrolyte leakage (EL), E) percentage low temperature seedling survivability-blup (LTSS-blup) and F) LTSS-logit (LTSS-logit). Correlations highlighted in blue are significant at *p*=0.01 and in orange at *p*=<0.0001. Correlation coefficients were calculated using the multivariate option, restricted maximum likelihood (REML) method.
